# Shallow-water zoantharians (Cnidaria, Hexacorallia) from the Central Indo-Pacific

**DOI:** 10.3897/zookeys.444.7537

**Published:** 2014-10-07

**Authors:** James D. Reimer, Angelo Poliseno, Bert W. Hoeksema

**Affiliations:** 1 Molecular Invertebrate Systematics and Ecology Laboratory, Faculty of Science, University of the Ryukyus, 1 Senbaru, Nishihara, Okinawa 903–0213, Japan; 2 Department of Marine Zoology, Naturalis Biodiversity Center, P.O. Box 9517, 2300 RA Leiden, The Netherlands; 3 Università Politecnica delle Marche, Via Brecce Bianche, 60131, Ancona, Italy

**Keywords:** Zoantharians, Indonesia, Indo-Pacific, biodiversity, coral reef, benthos

## Abstract

Despite the Central Indo-Pacific (CIP) and the Indonesian Archipelago being a well-known region of coral reef biodiversity, particularly in the ‘Coral Triangle’, little published information is available on its zoantharians (Cnidaria: Hexacorallia: Zoantharia). In order to provide a basis for future research on the Indo-Pacific zoantharian fauna and facilitate comparisons between more well-studied regions such as Japan and the Great Barrier Reef, this report deals with CIP zoantharian specimens in the Naturalis collection in Leiden, the Netherlands; 106 specimens were placed into 24 morpho-species and were supplemented with 88 in situ photographic records from Indonesia, the Philippines, and Papua New Guinea. At least nine morpho-species are likely to be undescribed species, indicating that the region needs more research in order to properly understand zoantharian diversity within the CIP. The Naturalis’ zoantharian specimens are listed by species, as well as all relevant collection information, and in situ images are provided to aid in future studies on zoantharians in the CIP.

## Introduction

Zoantharians (Cnidaria: Anthozoa: Hexacorallia: Zoantharia) are a common component of benthos in subtropical and tropical coral reef systems, with many zooxanthellate species found in shallow waters of both the Atlantic and Indo-Pacific Oceans. Nevertheless, common understanding of zoantharian species diversity is relatively poor when compared to the hard corals (Scleractinia). This lack of knowledge is due to a variety of reasons, including (1) high levels of intraspecific morphological variation hindering reliable identification (Burnett et al. 1997, Reimer et al. 2004), (2) problems in performing histological examinations owing to sand being incorporated in the body walls of many zoantharian species (Reimer et al. 2010), and (3) a confused taxonomic history as different researchers tried to properly classify and understand zoantharian diversity (Burnett et al. 1997, Reimer et al. 2004, Sinniger et al. 2005).

Despite these problems, an understanding of zoantharian diversity and their corresponding taxonomy have slowly become clearer as molecular techniques have been implemented into zoantharian research. The first molecular works of Burnett and co-workers (Burnett et al. 1994, 1995, 1997) combined with the ecological and descriptive works of Ryland (Ryland and Lancaster 2003, 2004) have led to more recent papers dealing with the molecular phylogeny of zoantharians (Reimer et al. 2004, 2012b, Sinniger et al. 2005, Swain 2010), resulting in a reassessment of zoantharian taxonomy (Fujii and Reimer 2011, 2013, Sinniger et al. 2013). Consequently, zoantharians are now perhaps the hexacorallian order for which the taxonomy most accurately reflects molecular phylogenetic understanding. However, whereas zoantharian supraspecific taxonomy and diversity is increasingly well understood, many problems remain at the species level (Reimer et al. 2007b), and total species diversity of zoantharians is still poorly known (Appeltans et al. 2012).

Recent work on zoantharians has focused on many regions of the Indo-Pacific, including Japan (Reimer 2010), Singapore (Reimer and Todd 2009), New Caledonia (Sinniger 2006), the Great Barrier Reef (Burnett et al. 1997, Reimer et al. 2011a) and Palau (Reimer et al. 2014a). In the center between these regions lies the central Indo-Pacific “Coral Triangle” (Hoeksema 2007), including parts or all of Malaysia, Indonesia, Brunei, the Philippines, and Papua New Guinea, the Solomons, and Timor Leste. This region is believed to harbor the highest species diversity in hard corals of the order Scleractinia (Hoeksema 2007, Veron et al. 2009, 2011), and it is believed that other coral reef organisms likely have similar diversity patterns (Roberts et al. 2002). Despite this, shallow-water zoantharian species within the Coral Triangle have only briefly been reported on in scientific literature and only a few publications exist (e.g. Den Hartog 1997, Sinniger et al. 2005, Di Camillo et al. 2010), and most information is made up of photographs in aquarium handbooks (Fosså and Nilsen 1998) and field guides (Colin and Arneson 1995, Gosliner et al. 1996, Erhardt and Knop 2005). Therefore, efforts to compare the regional zoantharian fauna of the Indo-Pacific are hampered by this almost complete lack of published scientific distribution information. Basic data on zoantharians from the Coral Triangle, such as species lists and distribution records, are critical to achieve a comprehensive understanding of Indo-Pacific zoantharian diversity.

The present study addresses this lack of Central Indo-Pacific (CIP) zoantharian data via examinations of specimen collections housed in Naturalis Biodiversity Center, Leiden, the Netherlands: RMNH (the former Rijksmuseum van Natuurlijke Historie) and ZMA (the former Zoologisch Museum van Amsterdam). These zoantharian collections are partly based on specimens from numerous surveys in Indonesia dating from the Snellius Expedition (1929–1930) to a recent Marine Biodiversity Workshop in Lembeh Strait (2012), with the large majority of these specimens collected from coral reef environments. Despite the presence of these large and scientifically valuable collections, no previous effort has been made to comprehensively catalogue or examine these historical collections for over 80 years, which could also serve as base-line material for studies on biotic change (Hoeksema et al. 2011). Here, for the first time, we report on the zoantharian specimens from Indonesia housed at Naturalis, and list shallow water species of the CIP, including specimen collection information. Our records are further enhanced by numerous *in situ* images from more recent fieldwork in Indonesia taken by the last author starting with the Snellius–II Expedition (1984–1985). Finally, we discuss the shallow water zoantharian diversity of CIP in relation to information from surrounding regions, and make recommendations for future zoantharian research in the region.

## Materials and methods

### Specimen collection

**Figure 1.**
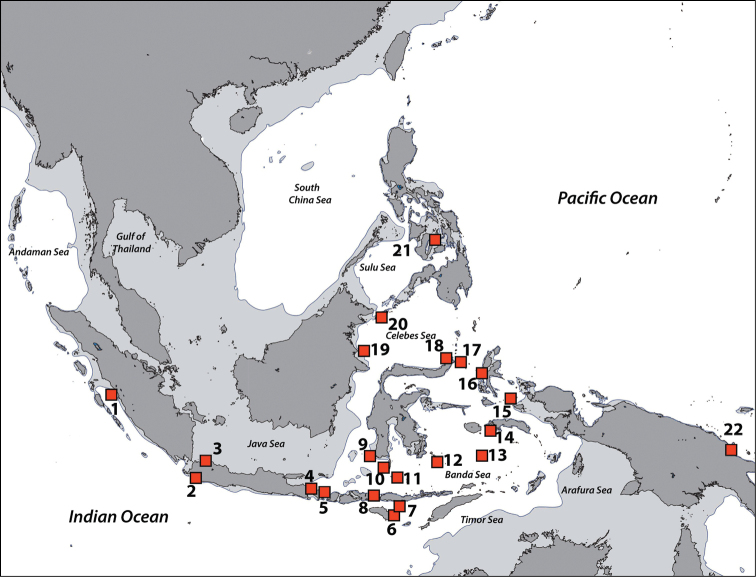
Sampling regions in this study. Note that the region numbers correspond with numbers given in text. **1** West Sumatra **2** Southwest Java **3** Thousand Islands, northwest Java, Java Sea **4** West Bali **5** East Bali **6** Northeast Sumba **7** South Flores **8** Komodo Island **9** Spermonde Archipelago, Southwest Sulawesi **10** Salayer Island, Southwest Sulawesi **11** Taka Bone Rate, Flores Sea **12** Tukang Besi Islands (Wakatobi), Southeast Sulawesi **13** Maisel Islands, Banda Sea **14** Ambon and Haruku, Moluccas **15** Bo Islands, Halmahera Sea **16** West Halmahera **17** Lembeh Strait, North Sulawesi **18** Bunaken, North Sulawesi **19** Berau Islands, East Kalimantan **20** Sulu Islands, Philippines **21** Cebu, Philippines **22** Madang, Papua New Guinea. Regions with no country names are in Indonesia. Oceanic names in italics. Dark grey: land masses. Light grey: continental flats.

Zoantharian specimens from the Naturalis collections in Leiden (RMNH + ZMA) were collected primarily from expeditions to the Indonesia region, starting with the Snellius Expedition (1929–1930). Our examinations showed 22 regions in which either specimens or photographic records were present. All specimen/record localities are shown in Figure 1 with location and reference details in Table 1.

**Table 1.** Overview of field surveys from which order Zoantharia specimens examined in this study were collected.

	Area	Year(s)	References	Remarks on locality and conditions of sample collecting
1	**West Sumatra**, Indonesia	1996	Jonker and Johan (1999: Figure 1)	Reefs off Padang and Siberut. Coral reef survey in collaboration with Bung Hatta University, Padang. Most reefs damaged, possibly as a result of blast fishing and red tide. Observer/collector: B.W. Hoeksema.
2	**Southwest Java**, Indonesia	1977	NA	Locality: Teluk Pelabuhan Ratu. Observer/collector: P.H. van Doesburg, RMNH.
3	**Northwest Java**, Indonesia	2005	Tuti and Soemodihardjo (2006): Annex 2 (157–161), Annex 5 (179)	Thousand Islands Expedition, off Jakarta, Java Sea. In collaboration with RCO–LIPI. Zoantharians were observed during a coral survey along an onhsore-offshore gradient.
4	**West Bali**, Indonesia	1998	Hoeksema and Putra (2000: Figure 1)	Coral biodiversity survey in collaboration with WWF Indonesia Marine Program.
5	**Eastern Bali**, Indonesia	1997, 1998	Hoeksema and Putra (2000: Figure 1)	Includes southeast Bali and Lombok Strait. Coral biodiversity surveys in collaboration with RCO–LIPI and WWF Bali Indonesia Marine Program.
		2001	Hoeksema and Tuti (2001: 12–15)	Includes southeast Bali and Lombok Strait. Coral biodiversity surveys in collaboration with RCO–LIPI and WWF Bali Indonesia Marine Program.
6	**Northeast Sumba**, Indonesia	1984	Van der Land and Sukarno (1986: 2.4–2.6, 3.5), Best et al. (1989: 108)	Indonesian – Dutch Snellius–II Expedition.
7	**South Flores**, Indonesia	1930	Boschma (1936: 24)	Snellius Expedition.
8	**Komodo Island**, Indonesia	1984	Van der Land and Sukarno (1986: 2.6–2.9, 2.19–2.20, 3.6), Best et al. (1989: 108)	Indonesian – Dutch Snellius–II Expedition.
9	**Spermonde Archipelago, South Sulawesi.** Indonesia	1980	Moll (1983: 26)	Coral reef surveys on reefs along onshore-offshore gradients. Observer/collector: H. Moll
		1984	Van der Land and Sukarno (1986: 2.22, 3.10), Best et al. (1989: 108)	Indonesian – Dutch Snellius–II Expedition.
		1984–1987	Hoeksema (2012a: Figure 1)	Coral reef surveys on reefs along onshore-offshore gradients.
		1993–1998	Hoeksema and Crowther (2011: Figure 1)	Coral reef surveys on reefs along onshore-offshore gradients.
10	**Salayer Island, S Sulawesi**, Indonesia	1984	Van der Land and Sukarno (1986: 2.12–2.17, 3.9), Best et al. (1989: 108)	Indonesian – Dutch Snellius–II Expedition.
11	**Taka Bone Rate (Tiger Is.)**, Indonesia	1984	Van der Land and Sukarno (1986: 2.11–2.12, 2.17–2.19, 3.8), Best et al. (1989: 108)	Indonesian – Dutch Snellius–II Expedition.
12	**Tukang Besi Is. (Wakatobi), SE Sulawesi**, Indonesia	1984	Van der Land and Sukarno (1986: 2.2–2.4, 3.4), Best et al. (1989: 108)	Indonesian – Dutch Snellius–II Expedition.
		2003	Pet-Soede and Erdmann (2004: 57, 117, 143)	Rapid Ecological Assessment (REA) Wakatobi National Park.
13	**Maisel Is., Banda Sea**, Indonesia	1984	Van der Land and Sukarno (1986: 2.2, 3.3), Best et al. (1989: 108)	Indonesian – Dutch Snellius–II Expedition.
14	**Moluccas (Ambon, Haruku)**, Indonesia	1930	Boschma (1936: 19–20)	Snellius Expedition.
		1984	Van der Land and Sukarno (1986: 2.1–2.2), Best et al. (1989: 108)	Indonesian – Dutch Snellius–II Expedition.
		1990	Strack (1993: 16–42)	Rumphius Biohistorical Expedition to Ambon.
		1996	Van der Land (1996)	Fauna Malesiana Marine Maluku Expedition.
15	**Bo Is., Halmahera Sea**, Indonesia	1930	Boschma (1936: 23)	Snellius Expedition.
16	**West Halmahera Sea**, Indonesia	2009	Hoeksema and Van der Meij (2010: 80–85)	Ekspedisi Widya Nusantara (E–Win): Ternate Expedition. Coral biodiversity survey B.W. Hoeksema.
		2009	Gittenberger et al. (2014: Figure 1)	Ekspedisi Widya Nusantara (E–Win): Ternate Expedition. Coral biodiversity survey B.W. Hoeksema.
17	**Lembeh Strait, North Sulawesi**, Indonesia	1994	Van der Land (1994: 7–9)	Fauna Malesiana Marine Sulawesi Expedition in collaboration with RCO–LIPI.
		2012	NA	Marine Biodiversity Workshop North Sulawesi in collaboration with RCO–LIPI and Universitas Sam Ratulang. Observer/collector: B.W. Hoeksema.
18	**Bunaken, North Sulawesi**, Indonesia	1994, 1998	NA	Fieldwork in collaboration with Universitas Sam Ratulang, Manado. Observer/collector: B.W. Hoeksema.
19	**Berau Islands, East Kalimantan**, Indonesia	2003	Hoeksema (2004: 57–60, Figure 1)	East Kalimantan Program. Coral biodiversity survey B.W. Hoeksema.
20	**Sulu Islands**, Philippines	1929	Boschma (1936: 8–9)	Snellius Expedition.
21	**Cebu, Bohol** Philippines	1976	NA	Fieldwork by M.L. Esmeno.
		1999	NA	Coral biodiversity survey B.W. Hoeksema during Cebu Strait Expedition in collaboration with San Carlos University, Cebu City.
22	**Madang, Bismarck Sea**, Papua New Guinea	1992	Hoeksema (1993: Figures 1–2)	Coral biodiversity survey in collaboration with Christensen Research Institute.

NA=not available.

Regions (numbers also referred to in species notes and in distributional maps, with names used hereafter in bold, and with representative publications included):

1. **West Sumatra**, Indonesia. Fieldwork by B.W. Hoeksema in collaboration with Dr. A. Kunzmann, Bung Hatta University, Padang, West Sumatra, in 1996–1997.
2. **Southwest Java**, Indonesia. Collections from Teluk Pelabuhan Ratu by Dr. P.H. van Doesburg, RMNH, in 1977.
3. Thousand Islands, off Jakarta, Java Sea, **northwest Java**, Indonesia. Expedition organized by the Research Center for Oceanography (RCO–LIPI) and Naturalis in 2005 (Tuti and Soemodihardjo 2006).
4. **West Bali**, Indonesia. Fieldwork by B.W. Hoeksema in collaboration with K.S. Putra of WWF Indonesia Marine Program in 1998 (Hoeksema and Putra 2000).
5. **East Bali** (including southeast Bali, Nusa Lembongan, Nusa Penida in Lombok Strait), Indonesia. Fieldwork by B.W. Hoeksema in collaboration with K.S. Putra of WWF in 1997 and 1998 (Hoeksema and Putra 2000). Expedition organized by the Research Center for Oceanography (RCO–LIPI), WWF Bali Indonesia Marine Program, and Naturalis in 2001 (Hoeksema and Tuti 2001).
6. **Northeast Sumba**, Indonesia. Indonesian – Dutch Snellius–II Expedition in 1984 (Van der Land and Sukarno 1986, Best et al. 1989).
7. **South Flores**, Indonesia. Snellius Expedition in 1929–1930 (Boschma 1936).
8. **Komodo Island**, Indonesia. Indonesian – Dutch Snellius–II Expedition (Van der Land and Sukarno 1986, Best et al. 1989).
9. **Spermonde Archipelago**, South Sulawesi, Indonesia. Snellius Expedition in 1929–1930 (Boschma 1936). Indonesian – Dutch Snellius–II Expedition in 1984 (Van der Land and Sukarno 1986, Best et al. 1989). Fieldwork by Dr. H. Moll in 1980 (Moll 1983). Fieldwork by B.W. Hoeksema around reefs along onshore-offshore gradients in 1984–1987 (Hoeksema 2012a) and in 1993–1998 (Hoeksema and Crowther 2011).
10. **Salayer Island**, South Sulawesi, Indonesia. Indonesian – Dutch Snellius–II Expedition in 1984 (Van der Land and Sukarno 1986, Best et al. 1989).
11. **Taka Bone Rate** (Tiger Islands), Flores Sea, Indonesia. Indonesian – Dutch Snellius–II Expedition in 1984 (Van der Land and Sukarno 1986, Best et al. 1989).
12. **Tukang Besi Islands** (Wakatobi), Southeast Sulawesi, Indonesia. Indonesian – Dutch Snellius–II Expedition in 1984 (Van der Land and Sukarno 1986, Best et al. 1989). Rapid Ecological Assessment (REA) Wakatobi National Park in 2003 (Pet–Soede and Erdmann 2004).
13. **Maisel Islands**, Banda Sea, Indonesia. Indonesian – Dutch Snellius–II Expedition in 1984 (Van der Land and Sukarno 1986, Best et al. 1989).
14. Ambon and Haruku, **Moluccas**, Indonesia. Snellius Expedition in 1929–1930 (Boschma 1936). Indonesian – Dutch Snellius–II Expedition in 1984 (Van der Land and Sukarno 1986, Best et al. 1989). Rumphius Biohistorical Expedition to Ambon in 1990 (Strack 1993). Fauna Malesiana Marine Maluku Expedition in 1996 (Van der Land 1996).
15. **Bo Islands**, Halmahera Sea, Indonesia. Snellius Expedition in 1929–1930 (Boschma 1936).
16. **West Halmahera Sea**, Indonesia. Ekspedisi Widya Nusantara (E–Win): Ternate Expedition in 2009, involving reefs on volcanic slopes and reefs around sand-cays (Hoeksema and Van der Meij 2010; Gittenberger et al. in press).
17. **Lembeh Strait**, North Sulawesi, Indonesia. Fauna Malesiana Marine Sulawesi Expedition organized by Research Center for Oceanography (RCO–LIPI) and Naturalis in 1994. Marine Biodiversity Workshop North Sulawesi organized by Research Center for Oceanography (RCO–LIPI), Universitas Sam Ratulang and Naturalis in 2012.
18. **Bunaken**, North Sulawesi, Indonesia. Fieldwork by B.W. Hoeksema in collaboration with Universitas Sam Ratulang, Manado, in 1994 and 1998.
19. **Berau Islands**, East Kalimantan, Indonesia. East Kalimantan Program in 2003 (Hoeksema 2004).
20. **Sulu Islands**, Philippines. Snellius Expedition in 1929–1930 (Boschma 1936).
21. **Cebu**, Philippines. Cebu Fieldwork by M. L. Esmeno in 1976. Strait Expedition organized by San Carlos University and National Museum of Natural History, Leiden in 1999.
22. **Madang**, Bismarck Sea, north coast of Papua New Guinea. Fieldwork by B.W. Hoeksema with Christensen Research Institute in 1992 (Hoeksema 1993).

### Specimen registration and identification

Examination of the registered (n=52) and unregistered zoantharian specimens (n=570) of the Naturalis collection showed that of a total 622 specimens, 105 were from Indonesia, with an additional four from the Philippines. Of these 109 specimens, 106 form the basis of this research, as we excluded three specimens that could not be conclusively identified as zoantharians. 88 photographic records of zoantharians specimens were also examined.

Although most species are from depths in the range of SCUBA (<40 m), we also included all *Epizoanthus illoricatus* Tischbierek, 1930 specimens, as although some specimens were from >40 m (and down to 190 m), the range of this species does extend into shallower (<40 m) depths. Additionally, three specimens of *Parazoanthus* collected by rectangular dredge from depths of 50–100 m were included in analyses. In this study, these 106 zoantharian specimens are collectively referred to as “shallow-water zoantharians”.

All unregistered specimens were newly registered into the Naturalis collection in the course of our research. All specimens, newly registered or not, were re-identified by the first author. A list of specimens, their collection information, and Naturalis (RMNH Coel) registration numbers are given within each species’ section. Descriptions of each species are given to aid in field and specimen identification, and are not formal taxonomic redescriptions.

Most zoantharian specimens were easily identifiable to genus level without microscopic examination. Species determinations were made consulting previous literature (listed with each species). However, many specimens were only identified to “confers with” (cf.) or “affinity” (aff.) levels. Asides from a few species (e.g. *Palythoa heliodiscus*), very few records of zoantharians had previously been formally reported from the CIP/Coral Triangle region. Given these reasons, we followed recent research (Burnett et al. 1994, 1995, 1997, Reimer et al. 2006a, 2007b, Sinniger 2006, Sinniger et al. 2010, Reimer and Todd 2009, Reimer 2010, Reimer et al. 2011a, Fujii and Reimer 2011) from neighboring regions and used *Zoanthus* and *Palythoa* species names for which numerous references, molecular data and/or accurate descriptions were available, unless specimens and/or images clearly did not match with previously published information.

Sizes of specimens are averages taken from measurements of 10 polyps per specimen, unless the specimen contained less than 10 polyps, in which case all non-damaged polyps were examined. For species’ dimensions, average dimensions were taken from the overall average of specimens, unless there were less than three specimens within a species. In such cases, dimensions are stated only as a range (minimum to maximum).

## Results

From specimen examination, the 106 Indonesian zoantharian specimens in the Naturalis collection supplemented with images were placed into 24 morphospecies, detailed below. Locations are in Indonesia unless otherwise noted, and all photographic images were taken by B.W. Hoeksema unless otherwise noted. Duplicate photographic images of the same species from the same site are counted as one record. Latitude and longitude are given when available.

### Specimens and species

Abbreviations: NA=not available.

#### Order Zoantharia Gray, 1832

##### Suborder Brachycnemina Haddon & Shackleton, 1891a

###### Family Zoanthidae Rafinesque, 1815

####### Genus *Acrozoanthus* Saville–Kent, 1893

######## 1. Acrozoanthus australiae

Taxon classificationAnimaliaZoanthariaZoanthidae

Saville–Kent, 1893

Figures 2A , 3

######### Specimens examined

(n=16). RMNH Coel 23405, Tg. Bengteng (=Galghoek), Ambon, Moluccas, depth = 3 to 4 m, collected November 10, 1990 by J.C. den Hartog; RMNH Coel 23406, outer bay, Ruhmatiga, Hitu, Ambon, Moluccas, depth = approx. 3 m, collected December 3, 1990 by J.C. den Hartog; RMNH Coel 23407, station 17, southeast side of Pombo Island, Ambon, Moluccas, depth = 6 m, collected November 17, 1994 by J.C. den Hartog; RMNH Coel 23408, west-northwest of Barrang Lompo, Spermonde Archipelago, South Sulawesi, depth = 1.5 to 4 m, collected December 23, 1994 by J.C. den Hartog; RMNH Coel 23409, entrance of harbor near light beacon, northwest of Gusung, Spermonde Archipelago, South Sulawesi, depth = 5 to 7 m, collected October 7, 1990 by J.C. den Hartog; RMNH Coel 23410, 7.5 km west of Makassar, Spermonde Archipelago, South Sulawesi (05°07'S, 119°20'E), depth = NA, collected May 31, 1994 by J.C. den Hartog; RMNH Coel 23411, west of Gusung (=Lae–Lae Keke) (=1 km northwest of Makassar), Spermonde Archipelago, South Sulawesi (05°07.5'S, 119°23'E), depth = NA, collected May 31, 1994 by J.C. den Hartog; RMNH Coel 24100, station MAL04, south coast northeast of Cape Hahurong, Ambon, Moluccas (03°47'S, 128°06'E), depth = 2 to 28 m, collected June 6, 1996 by J.C. den Hartog; RMNH Coel 40361, NNM–LIPI–WWF Expedition station BAL.16, southeast side of Pulau Serangan, Bali (08°44'48"S, 115°14'26"E), depth = to 10 m, collected April 6, 2001 by J. Goud; RMNH Coel 40549, Snellius–II Expedition station 4.011, reef edge west of Mai, Maisel Islands, Banda Sea (05°28'S, 127°31'E), depth = 1 to 30 m, collected September 7, 1984; RMNH Coel 40550, Snellius–II Expedition station 4.001, near Tawiri, Ambon Bay, Moluccas (03°42'S, 128°07'E), depth = approx. 1.5 to 8 m, collected September 4, 1984; RMNH Coel 40554, Snellius–II Expedition station 4.006, near Eri, Ambon Bay, Moluccas (03°45'S, 128°8'E), depth = approx. 1.5 to 5 m, collected September 4, 1984; RMNH Coel 40556, Snellius–II Expedition station 4.006, near Eri, Ambon Bay, Moluccas (03°45'S, 128°8'E), depth = approx. 1.5 to 5 m, collected August 29, 1984; RMNH Coel 40558, Snellius–II Expedition station 4.030, west coast of Binongko, Tukang Besi Islands, Banda Sea (05°55'S, 123°59'E), depth = approx. 3 to 4 m, collected September 10, 1984 by M. Slierings; RMNH Coel 40566, west side of Pulau Samalona, 7.5 km west of Makassar, Spermonde Archipelago, South Sulawesi (05°07'S, 119°20'E), depth = NA, collected February 18, 1994 by B.W. Hoeksema; RMNH Coel 40569, Fauna Malesiana Marine Sulawesi Expedition station SUL.06, Pantai Parigi, Pulau Lembeh, Selat Lembeh, North Sulawesi (01°28'N, 125°14'E), depth = 0 to 6 m, collected October 15, 1994 by M. Slierings.

######### Photographic records

(n=6). West side of Pulau Lae–Lae (05°08'05"S, 119°23'15"E), South Sulawesi, May 24, 1997; station MAL.19 (03°43'S, 128°03'E), Tanjune Batu Dua, east of Hatu, north coast of Ambon Bay, Moluccas, November 19, 1996; station MAL.22 (03°48'S, 128°06'E), southwest coast, east of Tunjung Nusanive, Ambon Bay, Moluccas, November 21, 1996; Nusa Penida, Lombok Strait, east Bali, May 26, 1998 (08°40'56"S, 115°28'56"E); northwest Pulau Samalona, Spermonde Archipelago, South Sulawesi (05°07'25"S, 119°20'10"E), January 12, 1997; western slope of Bone Lola shoal, Spermonde Archipelago, South Sulawesi (05°03'15"S, 119°21'15"E), April 22, 1998.

######### Description.

**Figure 2.**
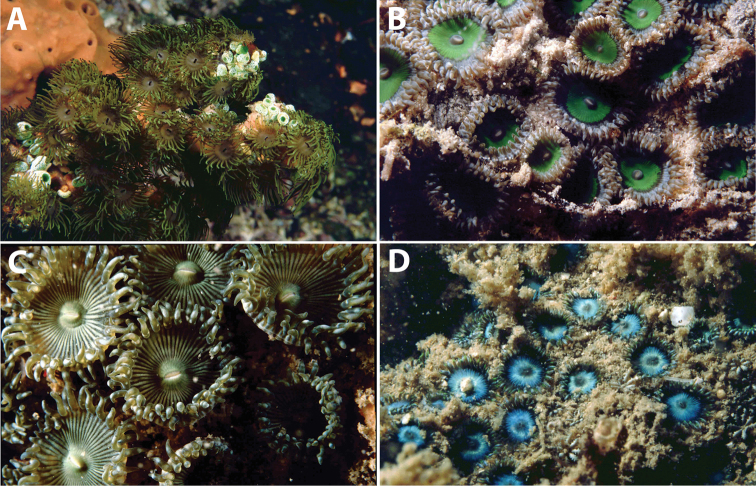
Images of *Acrozoanthus* and *Zoanthus* species from photographic records in this study. **A** *Acrozoanthus australiae* at Nusa Penida, Lombok Strait, east Bali, May 26, 1998 **B** *Zoanthus sansibaricus* at Station BER.26, northeast Buliulin (south of Samama Island), East Kalimantan, Berau Islands, October 15, 2003 **C** *Zoanthus* sp. at west side of Pulau Samalona, Spermonde Archipelago, South Sulawesi, September 16, 1997; and **D** *Zoanthus* sp. west of Gusung (=Pulau Lae–Lae Keke), Spermonde Archipelago, South Sulawesi, October 11 1997.

Non-incrusted zooxanthellate zoantharian that inhabits the outside of eunicid worm tubes (Haddon 1895), with a unique asexual form of “budding” (Ryland 1997). Easily recognizable as it is an epibiont on outside surface of eunicid worm tube, and has a reduced stoloniferous coenenchyme, long pale yellow-green or pale purple tentacles (n=approx. 40–50) with occasional fluorescent green markings and black tips, and light brown/purple to white ectoderm with similarly colored oral disks (Figure 2A). Preserved specimens in this study had polyps of average 6.0 mm in height (range 2.5–14 mm), 3.2 mm in width (range 2–5 mm) (n=8 specimens examined [RMNH Coel 40361, 40549, 40550, 40554, 40556, 40558, 40566, 40569], 10 polyps/specimen), and oral disks approximately 6 mm in diameter when expanded in situ (partially adapted from Reimer et al. 2011b). Specimen RMNH Coel 40566 had much larger polyps than other specimens (average height 10.6 mm, average width 4.3 mm), but this may be due to preservation in formalin as opposed to ethanol than to any phenotypic difference.

######### Distribution.

**Figure 3.**
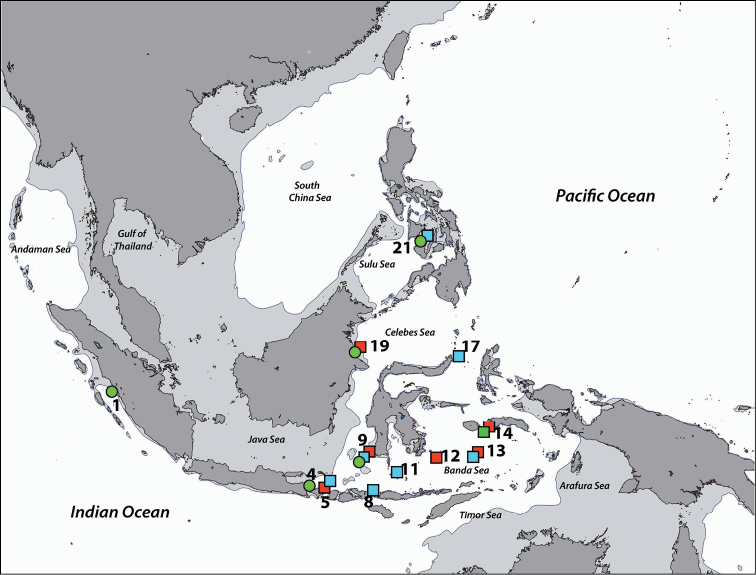
Distribution of *Acrozoanthus* and *Zoanthus* species from specimens and photographic records from this study. *Acrozoanthus australiae* specimens in red, *Zoanthus sansibaricus* in green, and *Zoanthus* sp. in blue. Region numbers correspond to locations given in species’ information. Boxes indicate presence of specimens (with or without photographic records), while circles indicate only photographic records. Overlapping symbols indicate the same region.

**Regions recorded in this study** (Figure 3). East coast of Bali (5), Spermonde Archipelago (9), Tukang Besi Islands (12), Maisel Islands (13), Moluccas (14), Lembeh Strait (17).

**Previous records.** Originally described from Australia, where it has been reported from both the coast of northern Queensland, and the region around Darwin in the Northern Territory. Subsequent records reported from North Sulawesi, Indonesia (Sinniger et al. 2005, Reimer et al. 2011b), and photographic records from Mactan Island, Philippines (Reimer et al. 2011b). Also reported from southern Taiwan (Reimer et al. 2011b) and at Ningaloo, Western Australia (Y. Irei and J.D. Reimer, unpubl. data).

######### Remarks.

This genus is positioned within the genus *Zoanthus* based on phylogenetic analyses (Reimer et al. 2011b).

####### Genus *Zoanthus* Lamarck, 1801

######## 2. Zoanthus sansibaricus

Taxon classificationAnimaliaZoanthariaZoanthidae

Carlgren, 1900

Figures 2B , 3

######### Specimens examined

(n=1). RMNH Coel 40476, Rumphius Biohistorical Expedition station 27, Leitimur, south coast, Hutumuri, Ambon, Moluccas, depth = intertidal, collected November 26, 1990 by M.S.S. Lavaleye.

######### Photographic records

(n=9). Southeast Siberut, West Sumatra (01°44'S, 99°15'E), December 15, 1996; east Menjangan Island, West Bali (08°05'25"S, 114°31'40"E), May 21, 1998; west Pulau Lumu Lumu, Spermonde Archipelago, South Sulawesi (04°58'30"S, 119°12'30"E), October 8, 1997; west Pulau Kudingareng Keke, Spermonde Archipelago, South Sulawesi (05°06'20"S, 119°17'03"E), May 29, 1997; northwest Pulau Barang Lompo, Spermonde Archipelago, South Sulawesi (05°02'35"S, 119°19'10"E), July 21, 1998; south Pulau Samalona, Spermonde Archipelago, South Sulawesi (05°07'45"S, 119°20'25"E), October 27, 1997; west Pulau Lae Lae Besar, Spermonde Archipelago, South Sulawesi (05°08'15"S, 119°23'10"E), November 12, 1997; northwest Pulau Lae Lae Keke, Spermonde Archipelago, South Sulawesi (05°07'10"S, 119°23'25"E), October 11, 1997; Station BER.26, northeast Buliulin (south of Samama Island), Berau Islands, East Kalimantan, (02°07'07"N, 118°20'32"E), October 15, 2003.

######### Description.

Can form colonies of up to 1 m^2^, but often forming much smaller colonies in cracks and small overhangs in intertidal and shallow waters (<5 m). with polyps well clear and free of the coenenchyme (“liberae”) (Pax 1910, Reimer et al. 2006b). Adult polyps 2–12 mm in diameter when open, up to 20 mm in length but usually shorter, particularly in locations with strong currents or waves. The sole specimen (RMNH Coel 40476) in this study has small polyps (height average 2.8 mm, range 2–5 mm; width average 2.4 mm, range 1.5–4 mm) within the reported range of this species. External polyp surface generally uniform, light to dark gray-blue with no significant markings or patterns. Tentacles 40–58, mesenteries 48–54. Wide variation in oral disk colors, patterns, and in colors of tentacles (Figure 2B) (Reimer et al. 2004, 2006a) (partially adapted from Reimer and Hickman 2009).

######### Distribution.

**Regions recorded in this study** (Figure 3). West Sumatra (1), West Bali (4), Spermonde Archipelago (9), Moluccas (14), Berau Islands (19).

**Previous records.** This species has previously been reported from Zanzibar (type locality), Singapore (Reimer and Todd 2009), Taiwan (Reimer et al. 2011c, 2013a), Palau (Reimer et al. 2014a), southern Japan (Reimer et al. 2004, 2006a, Kamezaki et al. 2013), and is considered to have a very wide Indo-Pacific distribution.

######### Remarks.

Based on its wide Indo-Pacific distribution, it is very likely that this zooxanthellate species is much more common within the CIP than reported here. One possible reason for the lack of records from the CIP is that this species is most commonly found in the intertidal zone, which is under-sampled during SCUBA surveys. However, this species is also found to depths of 52 m (Kamezaki et al. 2013), although below the shallow littoral zone it rarely forms colonies >100 polyps.

Additionally, as preserved specimens of *Zoanthus* are notoriously hard to identify to species level, the large number of unidentified *Zoanthus* specimens in this study undoubtedly include some *Zoanthus sansibaricus* colonies. This is also likely one important reason explaining the presence of comparatively more photographic records of this species in this study, as *in situ* identification of colonies with expanded oral polyps is easier than preserved specimen identification.

This species may be the same as *Zoanthus coppingeri* Haddon & Shackleton, 1891b from the Great Barrier Reef, Australia, based on molecular data (Reimer, data not shown), which has been reported to be a senior synonym of *Zoanthus jukesii* Haddon & Shackleton, 1891b, *Zoanthus macgillivrayi* Haddon & Shackleton, 1891b, *Zoanthus annae* Carlgren, 1937, *Zoanthus mantoni* Carlgren, 1937, *Zoanthus fraseri* Carlgren, 1937, all described from the Great Barrier Reef based on nematocyst data (Burnett et al. 1997).

######## 3. Zoanthus sp.

Taxon classificationAnimaliaZoanthariaZoanthidae

Figures 2C, D , 3

######### Specimens examined

(n=10). RMNH Coel 40360, NNM–LIPI–WWF Expedition station BAL.03, south of tidal channel, Palung Semawang, off Kesumasari Beach, Sanur, Bali (08°42'39"S, 115°16'09"E), depth to 5 m, collected by L. P. van Ofwegen and M. Slierings on March 31, 2001; RMNH Coel 40457, piers of harbor, Cebu City, Cebu, Philippines by M. L. Esmeno in 1976 (original label “specimen 196”); RMNH Coel 40516, Snellius–II Expedition station 27, west side of Bone Tambung, South Sulawesi (05°03'00"S, 119°15'45"E), depth = 1 m, collected October 23, 1980 by H. Moll; RMNH Coel 40537, Snellius–II Expedition station 4.139, reef flat edge south of Tarupa Kecil, northeast Taka Bone Rate (06°30'S, 121°08'E), depth = 30 m, collected September 25, 1984; RMNH Coel 40539, Snellius–II Expedition station 4.011, reef edge west of Mai, Maisel Islands, Banda Sea (05°28'S, 127°31'E), depth 1 to 30 m, collected September 7, 1984; RMNH Coel 40542, Snellius–II Expedition station 4.084, Selat Linta, east of Komodo I. (08°35'S, 119°34'E), depth = approx. 3 m, collected September 18, 1984; RMNH Coel 40551, Snellius–II Expedition station 4.079, Selat Linta, east of Komodo I. (08°35'S, 119°34.2'E), collected September 10, 1984; RMNH Coel 40560, Snellius–II Expedition station 4.096, northeast cape of Komodo I. (08°29'S, 119°34.1'E), from “shallow water”, collected September 20, 1984; RMNH Coel 40564, Fauna Malesiana Marine Sulawesi Expedition station SUL.08, channels between lava outflows, south of Tanjung Batuangus, Selat Lembeh, North Sulawesi (01°30'N, 125°15'E), depth 5 to 10 m, collected by M. Slierings on October 16 or 25, 1994; RMNH Coel 40565, Fauna Malesiana Marine Sulawesi Expedition station SUL.08, channels between lava outflows, south of Tanjung Batuangus, Selat Lembeh, North Sulawesi (01°30'N, 125°15'E), depth to 10 m, collected on October 16 or 25, 1994.

######### Photographic records

(n=3). West side of Pulau Lae–Lae, Spermonde Archipelago, South Sulawesi (05°08'05"S, 119°23'15"E), September 16, 1997; west side of Pulau Samalona, Spermonde Archipelago, South Sulawesi (05°07'25"S, 119°20'10"E), September 16, 1997; west of Gusung (=Pulau Lae–Lae Keke), Spermonde Archipelago, South Sulawesi (05°07.5'S, 119°23'E), October 11, 1997.

######### Description.

This group includes all *Zoanthus* spp. specimens that could not be identified to species level (n=10). Almost all of these specimens are ‘liberae’ or ‘intermediae’, with polyps rising out from the coenenchyme (see Pax 1910) (Figure 2C). One specimen, RMNH Coel 40560, is more ‘immersae’ with polyps only slightly protruding from the coenenchyme (average height 2.25 mm, width 2.0 mm, n=10). Overall, polyps for all specimens fit within the range of several described species, with an average for all specimens of a height of 6.7 mm (range 2–17 mm), and width of 3.3 mm (range 1.5–6 mm) (n=10 specimens). Thus, given the high variation within *Zoanthus* species, particularly polyp height based on microenvironment (Ong et al. 2013), and the lack of other diagnostic characteristics, for now these species cannot be identified to species level. Based solely on sizes, two specimens, RMNH Coel 40542 and 40565, have much larger polyps compared to the other specimens (height 10.3 mm, width 4.3 mm; height 10.8 mm, width 4.4 mm), but whether these are a separate species from other specimens or the size difference is due to fixation method in formalin is unknown.

######### Distribution.

**Regions recorded in this study** (Figure 3). Eastern Bali (5), Komodo (8), Spermonde Archipelago (9), Taka Bone Rate (11), Maisel Is. (13), Lembeh Strait. (17).

**Previous records.** NA.

######### Remarks.

This designation simply consists of all *Zoanthus* spp. specimens that could not be identified to species-level. It is likely this designation includes more than one species based on depths of specimens sampled. However, as preserved specimens were contracted (polyps closed) and many described *Zoanthus* spp. present no readily diagnostic external characters, identification to species level is not potentially possible without detailed molecular examination. Attempts at molecular identification also failed for these (and most other specimens), perhaps due to initial preservation in 10% seawater formalin for older specimens, or in ethanol with additives for newer specimens.

####### Genus *Isaurus* Gray, 1828

######## 4. Isaurus tuberculatus

Taxon classificationAnimaliaZoanthariaZoanthidae

Gray, 1828

Figures 4A, B , 5

######### Specimens examined

(n=3). RMNH Coel 40472, Rumphius Biohistorical Expedition station 27, Leitimur, south coast, Hutumuri, Ambon Bay, Moluccas (03°41'50"S, 128°17'00"E), intertidal under stones, collected on November 27, 1990 by J.C. den Hartog; RMNH Coel 40473, Rumphius Biohistorical Expedition station 27, Leitimur, south coast, Hutumuri, Ambon Bay, Moluccas (03°41'50"S, 128°17'00"E), intertidal under stones, collected on November 27, 1990 by J.C. den Hartog; RMNH Coel 40567, Fauna Malesiana Marine Sulawesi Expedition station SUL.04, bay south of Pulau Putus, Lembeh Strait, North Sulawesi (01°31'N, 125°16'E), depth approx. 1 to 2 m, on October 27, 1994 by J.C. den Hartog.

######### Photographic records.

NA.

######### Description.

**Figure 4.**
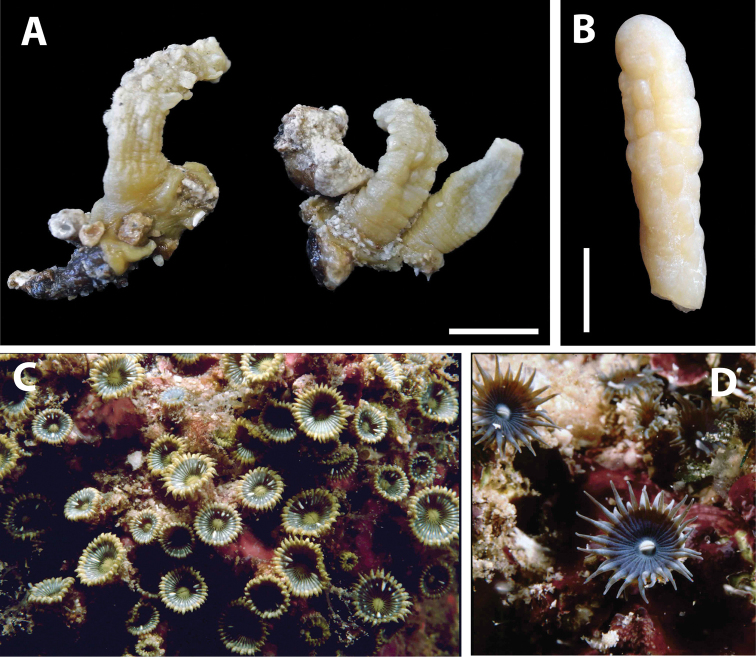
Images of *Isaurus* and *Neozoanthus* species from specimens and photographic records in this study. **A** *Isaurus tuberculatus* specimen RMNH Coel 40567 from Fauna Malesiana Marine Sulawesi Expedition station SUL.04, bay south of Pulau Putus, Lembeh Strait, North Sulawesi, depth approx. 1 to 2 m, on October 27, 1994 by J.C. den Hartog **B** *Isaurus tuberculatus* specimen RMNH Coel 40472 from Rumphius Biohistorical Expedition station 27, Leitimur, south coast, Hutumuri, Ambon Bay, Moluccas, intertidal under stones, collected on November 27, 1990 by J.C. den Hartog **C** *Neozoanthus* sp. at station WAK.13, southwest tip of Tolandono Island, REA Wakatobi National Park, Wakatobi, Southeast Sulawesi, on May 9, 2003; and **D** *Neozoanthus* sp. at Lembongan Bay, Nusa Lembongan, Lombok Strait, on May 19, 1998. Scales in **A** and **B** 1 cm.

Species in this genus are zooxanthellate, not incrusted, with a simple mesogleal sphincter muscle, and have non-erect, recumbent polyps that do not have lacunae or mesogleal canals, unlike *Zoanthus* species. *Isaurus tuberculatus* has tubercles on the exterior surface of polyps (=endodermal invagination) (Figures 4A, B). For detailed discussion of *Isaurus tuberculatus*, refer to Muirhead and Ryland (1985), with phylogenetic analyses in Reimer et al. (2008b).

Specimens examined in this study varied greatly in size from relatively large RMNH Coel 40567 (height 28–31 mm, width = 6–7 mm, n=2 polyps) to relatively small RMNH Coel 40473 (height average 10.6 mm, width average 2.9 mm, n=7 polyps). However, *Isaurus* polyps are known to vary greatly in size both between different colonies and within large colonies (Larson and Larson 1982; Muirhead and Ryland 1985; Reimer et al. 2008b). Furthermore, the two other valid Pacific *Isaurus* spp. asides from *Isaurus tuberculatus* are both very distinct from these specimens, and found in Fiji and southwestern Australia, respectively. Thus, the identity of these specimens as *Isaurus tuberculatus* is largely certain.

######### Distribution.

**Figure 5.**
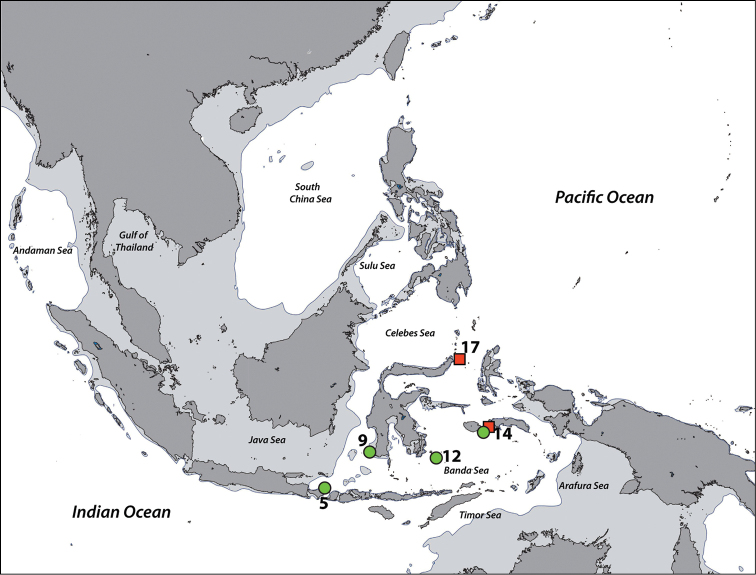
Distribution of *Isaurus* and *Neozoanthus* species from specimens and photographic records from this study. *Isaurus tuberculatus* specimens in red, and *Neozoanthus* sp. in green. Region numbers correspond to locations given in species’ information. Boxes indicate presence of specimens (with or without photographic records), while circles indicate only photographic records. Overlapping symbols indicate the same region.

**Regions recorded in this study** (Figure 5). Moluccas (14), Lembeh Strait (17).

**Previous records.** Originally described from the West Indies, this species is distributed throughout the subtropical and tropical Atlantic and Indo-Pacific (e.g. Muirhead and Ryland 1985), although populations in each ocean basin likely constitute different species (Reimer et al. 2008a). In the Indo-Pacific, it has previously been reported from the Great Barrier Reef, Fiji, Hawaii (summarized in Muirhead and Ryland 1985), and also from Indonesia (Sinniger et al. 2005), New Caledonia (Laboute and Richer de Forges 2004), and Japan (Reimer et al. 2008b).

######### Remarks.

As seen in previous studies (Reimer et al. 2008b), it appears from the low numbers of specimens here that *Isaurus* is either somewhat rare throughout its range, or cryptic in nature (e.g. well-camouflaged), resulting in few reports of this species.

###### Family Neozoanthidae Herberts, 1972

####### Genus *Neozoanthus* Herberts, 1972

######## 5. Neozoanthus sp.

Taxon classificationAnimaliaZoanthariaNeozoanthidae

Figures 4C, D , 5

######### Specimen regions.

NA.

######### Specimens examined.

NA.

######### Photographic records

(n=8). Gili Selang, eastern Bali (08°23'55"S, 115°42'30"E), on June 3, 1998; Lembongan Bay, Nusa Lembongan, Lombok Strait (08°40'25"S, 115°26'18"E), on May 19, 26, 27, 29, 1998 (4 records); Tanjung Taal, Nusa Lembongan, Lombok Strait (08°39'33"S, 115°26'37"E), on May 25, 1998; station WAK.22, north channel pass of Karang Koromaha, REA Wakatobi National Park, Wakatobi, Southeast Sulawesi (05°42'54"S, 124°10'53"E), on May 12, 2003; station WAK.13, southwest tip of Tolandono Island, REA Wakatobi National Park, Wakatobi, Southeast Sulawesi (05°46'35"S, 123°53'38"E), on May 9, 2003.

######### Description.

Unique among zoantharians, species in this genus have an endodermal sphincter with brachycnemic mesentery arrangement. Polyps are only partially incrusted, with the oral end of polyps lacking incrustation (Figures 4C, D). Phylogenetically, this genus is closely related to *Isaurus* (within family Zoanthidae). Zooxanthellate. Adapted from Herberts (1972), Reimer et al. (2012a).

######### Distribution.

**Regions recorded in this study** (Figure 5). Eastern Bali (5), Spermonde Archipelago (9), Tukang Besi Islands (12), Moluccas (14).

**Previous records.** Species of this genus have been reported from Madagascar (Herberts 1972), southern Japan (Reimer et al. 2011a, 2012a, 2013b), and the southern Great Barrier Reef (Reimer et al. 2011a, 2012a).

######### Remarks.

This genus was originally described from Madagascar with the type species *Neozoanthus tulearensis* Herberts, 1972. Subsequently, two species have been reported from Australia and Japan (Reimer et al. 2012a). As no specimens exist, it is impossible to determine if the Indonesian photographs constitute one or both of the species reported in Reimer et al. (2012a), or an as of yet undescribed species.

###### Family Sphenopidae Hertwig, 1882

####### Genus *Palythoa* Lamouroux, 1816

######## 6. Palythoa cf. mutuki

Taxon classificationAnimaliaZoanthariaSphenopidae

(Haddon & Shackleton, 1891b)

Figures 6A, B , 7

######### Specimens examined

(n=13): RMNH Coel 40458, harbor pier, Cebu City, Cebu, Philippines, collected in 1976 by M.L. Esmeno; RMNH Coel 40459, harbor pier, Cebu City, Cebu, Philippines, collected in 1976 by M.L. Esmeno; RMNH Coel. 40468, Rumphius Biohistorical Expedition station 29, Hitu, Ambon Bay, Ambon, Moluccas (03°38'05"S, 128°12'36"E), depth = intertidal, collected on November 28, 1990 by M.S.S. Lavaleye; RMNH Coel. 40470, Rumphius Biohistorical Expedition station 4, Leitimur, outer Ambon Bay, Wainitu, Moluccas (03°42'10"S, 128°09'15"E), depth = littoral on old shipwreck, collected on November 7–8, 1990 by H. Strack; RMNH Coel. 40475, Rumphius Biohistorical Expedition station 27, Leitimur, south coast, Hutumuri, Moluccas (03°41'50"S, 128°17'00"E), depth = intertidal, on November 26, 1990 by M.S.S. Lavaleye; RMNH Coel. 40514, Fauna Malesiana Maluku Expedition station MAL.15, Ambon Bay, south coast, cape west of Amahusu, Moluccas (03°44'S, 128°08'E), collected on November 16, 1996; RMNH Coel. 40528, Snellius–II Expedition station 4.096, northeast Komodo, Komodo (08°29'S, 119°34'E), depth = to 30 m, collected on October 26, 1984; RMNH Coel 40532, NNM–LIPI–WWF Bali–Lombok Strait 2001 Expedition station BAL.09, Loloan Batu Agung, Sanur, eastern Bali (08°43'31"S, 115°15'57"E), depth = 10 to 15 m, collected on April 3, 2001 by B.W. Hoeksema; RMNH Coel. 40540, Snellius–II Expedition station 4.010, near Tawiri, Ambon Bay, Moluccas (03°42'S, 128°07'E), depth = 1 to 5 m, collected on September 5, 1984; RMNH Coel. 40559, Snellius–II Expedition sta 4.012, north Pulau Mai, Maisel Islands, Banda Sea (05°28'S, 127°31'E), depth = 0 to 1.5 m, collected on 07.09.1984; RMNH Coel. 40561, Snellius–II Expedition station 4.133, east Pulau Tarupa Kecil, Taka Bone Rate (06°29'S, 121°08'E), depth = 11 m, collected on September 26, 1984; RMNH Coel. 40562, Snellius–II Expedition station 4.096, northeast Komodo, Komodo (08°29'S, 119°34'E), depth = to 30 m, collected on September 20, 1984; RMNH Coel. 40741, Rumphius Biohistorical Expedition station 11, Leitimur, Tanjung Nasaniwe, Moluccas (03°47'10"S, 128°05'20"E), depth = littoral, collected on November 12, 1990;

######### Photographic records

(n=2). Main coast, West Bali (08°06'50"S, 114°30'40"E), May 22, 1998; west Pulau Bone Batang, South Sulawesi, Spermonde Archipelago (05°01'00"S, 119°19'15"E), October 22, 1997.

######### Description.

Originally described from the Torres Strait, Australia, this species was redescribed in detail in Ryland and Lancaster (2003).

**Figure 6.**
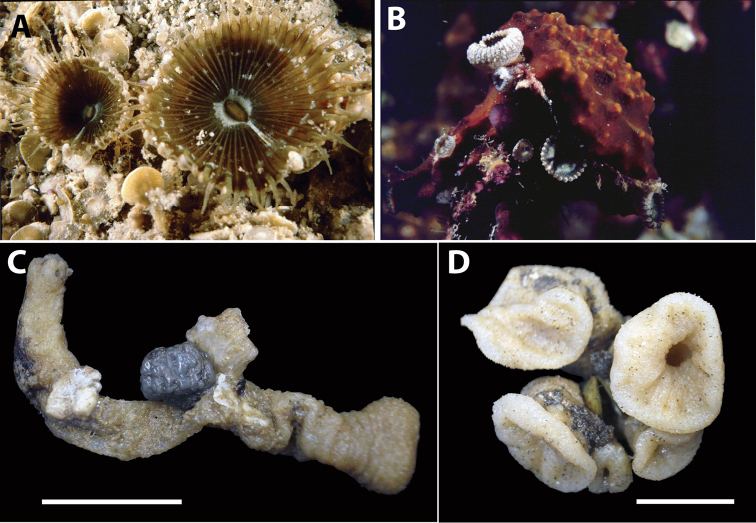
Images of Palythoa cf. mutuki from specimens and photographic records in this study. **A** Palythoa cf. mutuki at west Pulau Bone Batang, South Sulawesi, Spermonde Archipelago, October 22, 1997 **B** Palythoa cf. mutuki at main coast, West Bali, May 22, 1998 **C** *Palythoa* sp. specimen RMNH Coel 40508, Fauna Malesiana Maluku Expedition station MAL.13, west coast near Larike, Ambon, Moluccas, depth = 3 m, collected on November 15, 1996; and **D** *Palythoa* sp. specimen RMNH Coel 40512, Pelabuhan Ratu, Southwest Java, collected on October 13, 1977, by P.H. van Doesburg. Scales in **C** and **D** 1 cm.

Although all specimens in this grouping match with previously reported *Palythoa mutuki* based on sizes (average polyp height 9.6 mm, range 3–31 mm, average width 4.8 mm, range 2–8 mm, n=12 specimens) and overall morphology (‘intermediae’ or ‘liberae’ [Pax 1910]; visible capitulary ridges on closed polyps [Ryland and Lancaster 2003]) (Figure 6B), we have identified all specimens in this study as “cf.”. Recent work has shown the presence of more than two closely related species groups within *Palythoa mutuki* (Reimer et al. 2006b, 2013a) that are exceedingly difficult to distinguish without molecular data. For this reason, we have preliminarily assigned “cf.” to these specimens.

######### Distribution.

**Figure 7.**
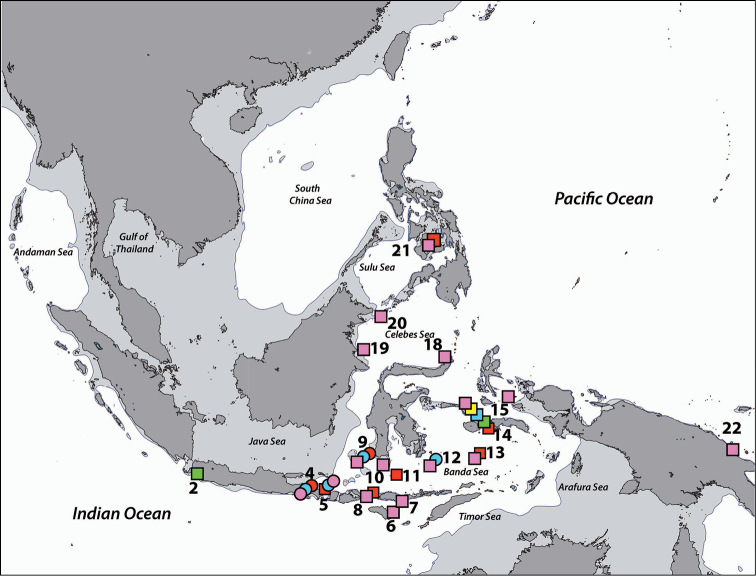
Distribution of *Palythoa* species from specimens and photographic records from this study. Palythoa cf. mutuki specimens in red, *Palythoa* sp. in green, Palythoa cf. heliodiscus in blue, Palythoa aff. tuberculosa in yellow, and *Palythoa tuberculosa* in pink. Region numbers correspond to locations given in species’ information. Boxes indicate presence of specimens (with or without photographic records), while circles indicate only photographic records. Overlapping symbols indicate the same region.

**Regions recorded in this study** (Figure 7). West Bali (4), eastern Bali (5), Komodo Island (8), Spermonde Archipelago (9), Taka Bone Rate (11), Maisel Islands (13), Moluccas (14), Cebu (21).

**Previous records.** Ryland and Lancaster (2003) in their treatment of *Palythoa mutuki* also mentioned records from Fiji, and synonymized records of other species from Tuvalu (*Gemmaria willeyi* Hill & Whitelegge, 1898), eastern Australia (*Gemmaria arenacea* Wilsmore, 1909; *Palythoa yongei* Carlgren, 1937; *Palythoa australiensis* Carlgren, 1950) and Singapore (*Palythoa singaporensis* Pax & Müller, 1956) with this species. However, asides from the specimens directly examined by Ryland and Lancaster, there is much confusion over the true identity of these species. For example, Ryland and Lancaster (2003) themselves state that *Gemmaria willeyi* is likely a *Zoanthus* species based on the figures in the original description. Ryland and Lancaster state “Probably only the use of genetic methods, so successfully applied by Burnett *et al.* (1997), will settle identities over wide geographic areas”.

However, in the Pacific, records of this species with phylogenetic confirmation have previously been reported from the Great Barrier Reef in Australia (Burnett et al. 1997), Singapore (Reimer and Todd 2009), to the south Pacific coast of Japan (e.g. Reimer et al. 2006b, 2007b), New Caledonia (Sinniger 2006), and across to the Galapagos (Reimer and Hickman 2009), and thus it is known that this species has a very wide Indo-Pacific distribution.

######### Remarks.

This species is likely common in Indonesia as in other regions such as Okinawa (Irei et al. 2011) and Taiwan (Reimer et al. 2011c). However, species delineation in *Palythoa* is confused due to the close phylogenetic relationships between *Palythoa mutuki*, *Palythoa tuberculosa*, and some other undescribed species, and a potential reticulate evolutionary history (Reimer et al. 2007b, Shiroma and Reimer 2010, M. Mizuyama and J.D. Reimer unpubl. data). Furthermore, distinguishing *Palythoa mutuki*, from other, more distantly related species such as *Palythoa heliodiscus* based solely on morphology is often difficult (Ryland and Lancaster 2003). For this study, we have included all “*Palythoa mutuki*-like” specimens as one species group for convenience, although it is likely the specimens will encompass more than one species once the taxonomy of this genus is clarified.

######## 7. Palythoa sp.

Taxon classificationAnimaliaZoanthariaSphenopidae

Figures 6C, D , 7

######### Specimens examined

(n=2): RMNH Coel 40508, Fauna Malesiana Maluku Expedition station MAL.13, west coast near Larike, Ambon, Moluccas (03°43'S, 127°56'E), depth = 3 m, collected on November 15, 1996; RMNH Coel 40512, Pelabuhan Ratu, southwest Java (07°01'N, 106°34'E), collected on October 13, 1977, by P.H. van Doesburg.

######### Photographic records.

NA.

######### Description.

This group consists of two specimens that do not clearly fit with previously described *Palythoa* species. Both specimens have dimensions very different from other *Palythoa* specimens reported here; whether this is due to unusual fixation or relaxation methods, or to true phenotypic differences is unknown.

RMNH Coel 40508 (Figure 6C) has very long ‘liberae’ polyps (average 23.6 mm height, n=4 polyps) that are more robust (average 5 mm, n=4 polyps) than seen in *Palythoa heliodiscus*, but with almost no development of the coenenchyme, unlike as in *Palythoa mutuki* or other closely related species. As well, this specimen is from 3 meters depth, a shallower depth than usually seen for *Palythoa heliodiscus*.

RMNH Coel 40512 (Figure 6D) is a small ‘intermediae’ colony consisting of four polyps that are squat and robust (average width 8.3 mm, n=3 polyps, height approximately same as width) with large oral discs (average 12 mm in diameter, n=3 polyps) with no tentacles visible and a large oral opening.

######### Distribution.

**Regions recorded in this study** (Figure 7). Southwest Java (2), Moluccas (14).

**Previous records.** NA.

######### Remarks.

The morphology of these specimens do not clearly match any described species from the central Indo-Pacific. In particular, specimen RMNH Coel 40512 is different than any other zoantharian previously observed by the first author. However, it is unknown if fixation has resulted in degradation of fine scale structures (e.g. tentacles, which are absent), but the specimen is clearly a zoantharian due to sand encrustation in body wall.

######## 8. Palythoa cf. heliodiscus

Taxon classificationAnimaliaZoanthariaSphenopidae

(Ryland & Lancaster, 2003)

Figures 7 , 8

######### Specimens examined

(n=2). RMNH Coel 40504, Fauna Malesiana Maluku Expedition station MAL.12, north coast near Morela, Ambon, Moluccas (03°33'S, 128°12'E), depth = 35 m, collected on November 13, 1996; RMNH Coel. 40513, Rumphius Biohistorical Expedition station 24, south Seri Bay, Ambon, Moluccas (03°34'50"S, 128°09'45"E), depth = 12 m, November 22, 1990.

######### Photographic records

(n=13). Pulau Ular, off Padang, West Sumatra (01°07'05"S, 100°20'02"E), December 16, 1996; Pemuteran, West Bali (08°11'20"S, 114°50'30"E), May 23, 1998; Tulamben, eastern Bali (08°16'26"S, 115°35'28"E), July 12, 1997; Nusa Lembongan, Lombok Strait (08°40'S, 115°26'E), May 29, 1998; west side Pulau Samalona, Spermonde Archipelago, South Sulawesi (05°07'25"S, 119°20'10"E), November, 1984; northwest side Pulau Samalona, Spermonde Archipelago, South Sulawesi (05°07'25"S, 119°20'10"E), November 23, 1997; northwest Kudingareng Keke, Spermonde Archipelago, South Sulawesi (05°06'15"S, 119°17'10"E), August 6, 1997; west side Pulau Badi, Spermonde Archipelago, South Sulawesi (04°58'06"S, 119°16'57"E), November 1, 1994; REA Wakatobi National Park station WAK.18, southwest Pulau Binongko, Southeast Sulawesi, Wakatobi, Tukang Besi Islands (05°59'48"S, 124°02'55"E), May 10, 2003; REA Wakatobi National Park station WAK.22, north channel pass of Karang Koromaha, Southeast Sulawesi, Wakatobi, Tukang Besi Is. (05°42'54"S, 124°10'53"E), May 12, 2003; Fauna Malesiana Maluku Expedition station MAL.12, north coast near Morela, Ambon (03°33'S, 128°12'E), November 13–14, 1996; East Kalimantan–Berau Expedition station BER.03, south side of Pulau Derawan, East Kalimantan (02°17'03"N, 118°14'49"E), October 16, 2003; Christensen Research Institute, Madang, Papua New Guinea (05°09'30"S, 145°48'10"E), June 1992.

######### Description.

This zooxanthellate species was described in detail recently by Ryland and Lancaster (2003). Superficially similar in appearance to *Palythoa mutuki*, externally the species can be distinguished by its short tentacles (length <20% of oral disk) and subtidal distribution, compared to primarily intertidal *Palythoa mutuki*, which also has longer tentacles (~45% of oral disk) (Ryland and Lancaster 2003).

Sizes of specimens agree well with specimens seen in other localities (average polyp heights 11.3 mm and 17.0 mm for each specimen, range 7–20 mm; average width 3.9 mm and 4.4 mm for each specimen, range 3.5–5.5 mm; n=2 specimens of 8 and 5 polyps, respectively). Depth of collected specimens (12 and 35 m) also fits well with the description of this species as primarily subtidal in the original description, and from data in Okinawa, Japan (e.g. Reimer 2010).

######### Distribution.

**Regions recorded in this study** (Figure 7). West Bali (4), eastern Bali (5), Spermonde Archipelago (9), Tukang Besi Islands (12), Moluccas (14).

**Previous records.** *Palythoa heliodiscus* has been reported from Australia (Ryland and Lancaster 2003) and is likely widespread in the Indo-Pacific (Ryland and Lancaster 2003 and references within), as well as Japan (Reimer et al. 2006b), Palau (Reimer et al. 2014a), while *Palythoa toxica* Walsh & Bowers, 1971 has been reported from Hawai’i.

######### Remarks.

**Figure 8.**
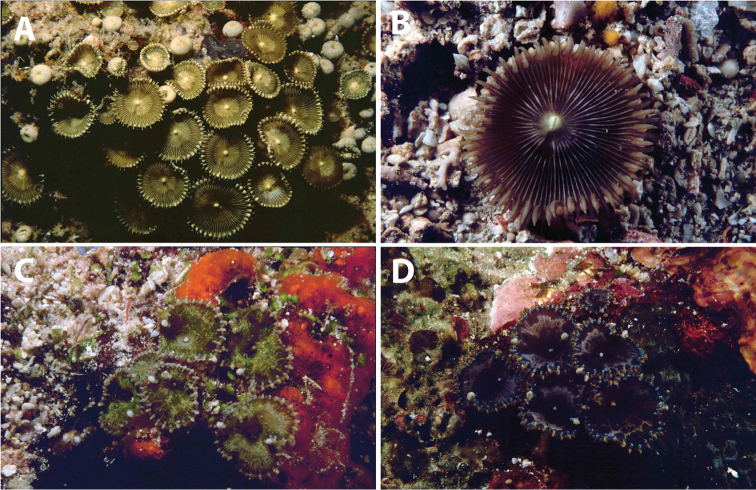
Images of Palythoa cf. heliodiscus from photographic records in this study. **A** Palythoa cf. heliodiscus at the northwest side of Pulau Samalona, Spermonde Archipelago, South Sulawesi, November 23, 1997 **B** Palythoa cf. heliodiscus at the south side of Pulau Derawan, East Kalimantan, October 16, 2003 **C** Palythoa cf. heliodiscus at REA Wakatobi National Park station WAK.22, north channel pass of Karang Koromaha, Southeast Sulawesi, Wakatobi, Tukang Besi Is., May 12, 2003; and **D** Palythoa cf. heliodiscus at REA Wakatobi National Park station WAK.18, Southwest Pulau Binongko, Southeast Sulawesi, Wakatobi, Tukang Besi Islands, May 10, 2003.

We have identified all specimens here as “cf.” as in situ images (Figure 8) there are two different morphotypes. One morphotype matches with *Palythoa heliodiscus*, with a brown oral disk with no patterns (Figures 8A, B), while the other morphotype’s polyps have either green or purple oral disks with various semi-irregular patterns, as well as blue/gray or light orange tentacles (Figures 8C, D). Based on data from Okinawa and Australia, both of these morphotypes are almost identical asides from the oral disk coloration and small but consistent differences in ITS–rDNA (T. Nishimura and J.D. Reimer, unpubl. data) that may be either intraspecific or interspecific. Thus, it is still uncertain if the green/purple morphotype is an undescribed species or not (Reimer et al. 2014a).

Furthermore, the overall morphology of the green/purple morphotype closely resembles *Palythoa toxica* from Hawai’i, and whether these Indonesian specimens are *Palythoa toxica* or *Palythoa heliodiscus*, and if these two species are synonyms needs to be ascertained before any formal description occurs. In situ images and further DNA sequences are therefore needed from future specimens.

######## 9. Palythoa aff. tuberculosa

Taxon classificationAnimaliaZoanthariaSphenopidae

(Esper, 1805)

Figures 7 , 9A

######### Specimens examined

(n=1). RMNH Coel 40521, Snellius Expedition, Pulau Haroekoe, east of Ambon, Ambon, Moluccas, collected on May 03–07, 1930.

######### Photographic records.

NA.

######### Description.

**Figure 9.**
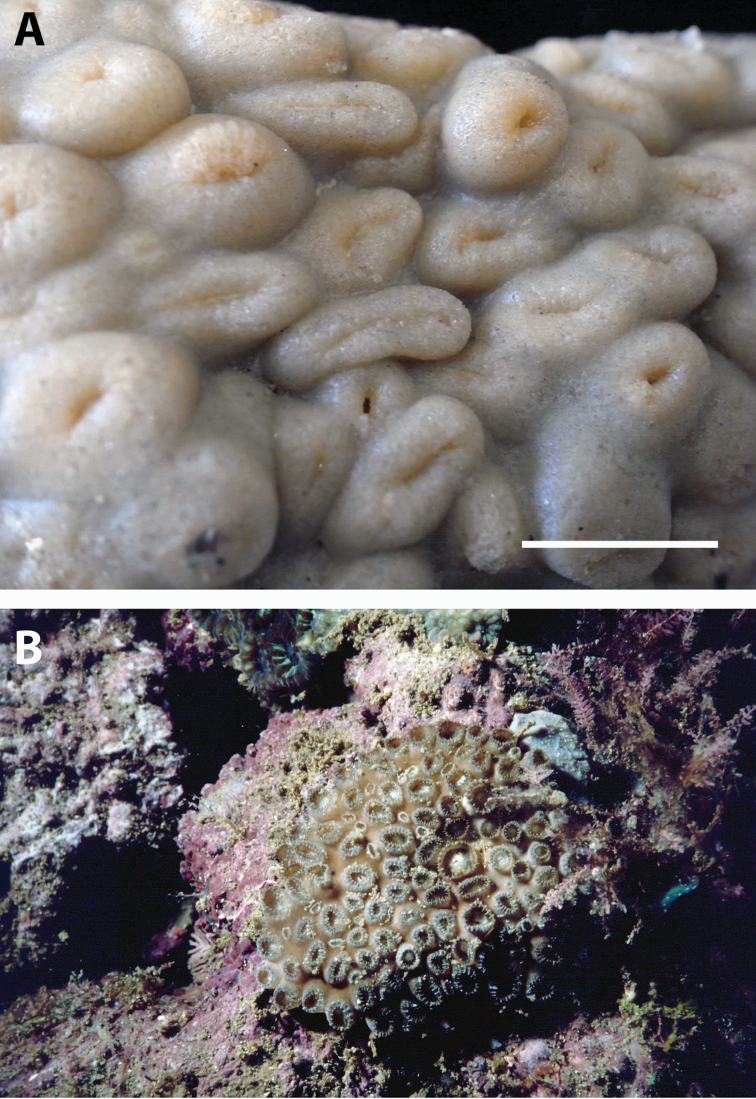
Images of *Palythoa tuberculosa* and Palythoa aff. tuberculosa from specimens and photographic records in this study. **A** Palythoa aff. tuberculosa specimen RMNH Coel 40521, Snellius Expedition, Sulu Islands, Philippines, collected on September 11–17, 1930; and **B** *Palythoa tuberculosa* at Madang, Papua New Guinea, June 1992. Scale in **A** 1 cm.

This specimen superficially resembles zooxanthellate *Palythoa* sp. yoron sensu Shiroma and Reimer (2010) with its very well developed coenenchyme and ‘intermediae–immersae’ morphology (Figure 9B). However, there are some differences between this specimen and *Palythoa* sp. yoron from Okinawa. The current specimen consists of two large portions of colonies consisting of >50 polyps, while *Palythoa* sp. yoron usually is found in very small colonies of <10 polyps. As well, *Palythoa* sp. yoron consists of a very well developed coenenchyme from which all individual polyps partially emerge, while the current specimen appears to consist more of large robust polyps that have merged together at many locations, but not at others, giving the specimen the appearance of *Palythoa tuberculosa* from the top, and often of *Palythoa mutuki* from side angles. On the other hand, *Palythoa* sp. yoron has an appearance, although intermediate between *Palythoa tuberculosa* and *Palythoa mutuki*, unique to and of itself. Polyps’ height (when not merged) is approximately 7.0 mm, and average width is 7.3 mm (n=10 polyps). Thus, for now, this specimen is identified as Palythoa aff. tuberculosa. For details on *Palythoa tuberculosa*, refer to the relevant species section below.

######### Distribution.

**Regions recorded in this study** (Figure 7): Moluccas (14).

**Previous records:** NA.

######### Remarks.

This specimen is unlike any other previous specimen observed in the field or museums by the first author. Unfortunately, as it was collected in 1930, attempts to acquire utilizable DNA sequences able to distinguish this specimen’s affinity were unsuccessful, and identification was made on gross morphology alone.

######## 10. Palythoa tuberculosa

Taxon classificationAnimaliaZoanthariaSphenopidae

Esper, 1805

Figures 7 , 9B

######### Specimens examined

(n=31). RMNH Coel 40465, Rumphius Biohistorical Expedition station 11, Leitimur, Tanjung Nasaniwe, Ambon, Moluccas (03°47'10"S, 128°05'20"E), depth = 2–5 m, collected on November 12, 1990; RMNH Coel 40466, Rumphius Biohistorical Expedition station 30, Hitu, Baguala Bay, Suli, Ambon, Moluccas (03°37'40"S, 128°17'50"E), collected on November 29, 1990; RMNH Coel 40467, Rumphius Biohistorical Expedition station 15, Hitu, Baguala Bay, 0.5 km west of Tial, Ambon, Moluccas (03°38'20"S, 128°19'40"E), depth = 2 m, collected on November 13–14, 1990; RMNH Coel 40471, Rumphius Biohistorical Expedition station 4, Leitimur, Ambon Bay, outer bay, Wainitu (near Ambon City), Ambon, Moluccas (03°42'10"S, 128°09'15"E), littoral on old shipwreck, collected on November 7–8, 1990 by H. Strack; RMNH Coel 40474, Rumphius Biohistorical Expedition station 27, Leitimur, south coast, Hutumuri, Ambon, Moluccas (03°41'50"S, 128°17'00"E), depth = 1 to 3 m, collected on November 27, 1990 by J.C. den Hartog; RMNH Coel 40505, south side of Barang Lompo, Spermonde Archipelago, South Sulawesi (05°03'23"S, 119°19'45"E), depth = 18 m, collected on October 18, 1980, by H. Moll; RMNH Coel 40511, west side of Pulau Samalona, Spermonde Archipelago, South Sulawesi (05°07'25"S, 119°20'10"E), depth = 2.5 m, collected on September 4, 1980 by H. Moll; RMNH Coel. 40517, west side of Pulau Samalona, Spermonde Archipelago, South Sulawesi (05°07'25"S, 119°20'10"E), depth = 2.5 m, collected on September 4, 1980 by H. Moll; RMNH Coel 40519, Snellius Expedition, Rumah Fija, Bo Islands, Halmahera Sea, collected on October 7, 1930; RMNH Coel 40522, Snellius Expedition, Sulu Islands, Philippines, collected on September 11–17, 1930; RMNH Coel 40523, Snellius Expedition, probably Indonesia, no locality data; RMNH Coel 40524, Snellius–II Expedition station 4.011, reef edge west of Mai, Maisel Islands, Banda Sea (05°28'S, 127°31'E), depth = 1–30 m, collected on September 7, 1984; RMNH Coel 40526, Snellius–II Expedition station 4.030, west coast of Pulau Binongko, Southeast Sulawesi, Tukang Besi Islands, Wakatobi (05°55'S, 123°59'E), depth approx. 2 m, September 10, 1984; RMNH Coel 40527, Snellius–II Expedition station 4.030, west coast of Pulau Binongko, Southeast Sulawesi, Tukang Besi Islands, Wakatobi (05°55'S, 123°59'E), depth approx. 0.5 m, September 10, 1984; RMNH Coel 40529, Snellius–II Expedition station 4.030, west coast of Pulau Binongko, Southeast Sulawesi, Tukang Besi Islands, Wakatobi (05°55'S, 123°59'E), depth approx. 8 m, September 10, 1984; RMNH Coel 40530, Snellius–II Expedition station 4.071, Slawi Bay, east Komodo, Komodo (08°34'30"S, 119°31'18"E), depth sublittoral, collected on September 17, 1984; RMNH Coel 40531, Snellius–II Expedition station 4.030, west coast of Pulau Binongko, Southeast Sulawesi, Tukang Besi Islands, Wakatobi (05°55'S, 123°59'E), depth approx. 3 to 4 m, September 10, 1984; RMNH Coel 40534, Snellius–II Expedition station 4.169, reef north of Pulau Bahuluang, Southwest Salayer, Salayer Island, South Sulawesi (06°27'S, 120°26'E), collected on September 30, 1984; RMNH Coel 40535, Snellius–II Expedition station 4.059, off Melolo, northeast Sumba (09°52'30"S, 120°40'18"E), collected on September 14, 1984; RMNH Coel 40541, Snellius–II Expedition station 4.006, Ambon Bay near Eri, Ambon, Moluccas (03°45'S, 128°08'E), depth approx. 3 m, collected on August 29, 1984; RMNH Coel 40543, Snellius–II Expedition station 4.006, Ambon Bay near Eri, Ambon, Moluccas (03°45'S, 128°08'E), depth = 0 to 10 m, collected on August 29, 1984; RMNH Coel 40548, Snellius–II Expedition station 4.052, east of Melolo, northeast Sumba (09°55'S, 120°45'E), depth approx. 3 m, collected on September 13, 1984; RMNH Coel 40552, Snellius–II Expedition station 4.048, east of Melolo, northeast Sumba (09°54'00"S, 120°43'30"E), depth = 12 m, collected on September 14, 1984; RMNH Coel 40553, Snellius–II Expedition station 4.096, northeast cape, Komodo (08°29'S, 119°34'E), depth to 30 m, collected on September 20, 1984; RMNH Coel 40555, Snellius–II Expedition station 4.096, northeast cape, Komodo (08°29'S, 119°34'E), depth to 30 m, collected on September 20, 1984; RMNH Coel 40557, Snellius–II Expedition station 4.096, northeast cape, Komodo (08°29'S, 119°34'E), depth = “shallow water”, collected on September 20, 1984; RMNH Coel 40568, northwest of Pulau Kapoposang, Spermonde Archipelago, South Sulawesi (04°41'40"S, 118°54'55"E), collected on May 2, 1998 by B.W. Hoeksema; RMNH Coel 40769, Snellius Expedition, Eude, South Flores, collected on March 6–8, 1930; RMNH Coel 40770, Snellius Expedition, Maratua, Berau Islands, East Kalimantan, collected on October 14–17, 1930; RMNH Coel 40771, Snellius Expedition, Maratua, Berau Islands, East Kalimantan, collected on October 14–17, 1930; RMNH Coel 40772, Snellius–II Expedition station 4.006, Ambon Bay near Eri, Ambon, Moluccas (03°45'S, 128°08'E), depth = 0 to 10 m, collected on August 29, 1984.

######### Photographic records

(n=12). Pemuteran, West Bali (08°08'S, 114°41'E), May 20, 1998; Pemuteran, West Bali (08°08'S, 114°41'E), May 23, 1998; Napoleon Reef, West Bali (08°08'S, 114°41'E), May 20, 1998; Nusa Lembongan, Lombok Strait, East Bali, July 13, 1997; Nusa Lembongan, Lombok Strait, east Bali, July 19, 1997; Nusa Lembongan, Lombok Strait, east Bali, May 26, 1998; south of Pulau Samalona, Spermonde Archipelago, South Sulawesi (05°07'45"S, 119°20'25"E), October 27, 1997; northwest Pulau Samalona, Spermonde Archipelago, South Sulawesi (05°07'25"S, 119°20'10"E), November 25, 1997; Fauna Malesiana Maluku Expedition station MAL.12, north coast near Morela, Ambon, Moluccas November 13, 1996; North Sulawesi, Bunaken, (01°36'N, 124°47'E), April 9, 1996; Cebu, Philippines, November 21, 1998; Madang, Papua New Guinea, June 1992.

######### Description.

This zooxanthellate species was originally described from India (Esper 1805), and subsequently redescribed utilizing specimens from the Red Sea (Klunzinger 1877). Recent work by Hibino et al. (2013) indicates the species may include some junior synonyms, and has a wide distribution across the subtropical and tropical Indo-Pacific. Polyps are embedded within a well-developed coenenchyme (‘immersae’, Pax 1910), and colonies vary in color from fluorescent green-yellow to dark brown or even ochre (Figure 9A).

Specimens in this study averaged 4.7 mm in polyp diameter (n=29 specimens), ranging from 2 to 8 mm. One specimen, RMNH Coel 40553, was notable for its very small polyps (average diameter 2.4 mm, n=10 polyps). Other colonies ranged from 3.1 to 6.5 mm in average diameter, similar to previous reported sizes. All specimens were ‘immersae’. Generally, morphology fit well within the accepted range of *Palythoa tuberculosa* (see Table 1 in Hibino et al. 2013), although some specimens’ polyps were somewhat smaller than previously observed. These smaller sizes may also be partly due to preservation methods.

######### Distribution.

**Regions recorded in this study** (Figure 7). West Bali (4), east Bali (5), northeast Sumba (6), south Flores (7), Komodo (8), Spermonde Archipelago (9), Salayer Island (10), Tukang Besi Islands (12), Maisel Islands (13), Moluccas (14), Bo Islands (15), Bunaken (18), Berau Islands (19), Sulu Islands (20), Cebu (21), Madang (22).

**Previous records.** This species has been phylogenetically confirmed as distributed over the entire subtropical and tropical Indo-Pacific, from at least the Red Sea to Singapore (Reimer and Todd 2009), Taiwan (Reimer et al. 2011c), Japan (e.g. Reimer et al. 2006a), New Caledonia (Sinniger 2006), and the Galapagos Islands (Reimer and Hickman 2009).

######### Remarks.

It is highly likely this species is the senior synonym of *Palythoa caesia* Dana, 1846 (Hibino et al. 2013), described from Fiji and commonly reported from Australia (Burnett et al. 1997). This species is also part of the *Palythoa tuberculosa*–*Palythoa mutuki* species complex (Reimer et al. 2007b, M. Mizuyama and J.D. Reimer unpubl. data).

####### Genus *Sphenopus* Steenstrup, 1856

######## 11. Sphenopus marsupialis

Taxon classificationAnimaliaZoanthariaSphenopidae

(Gmelin, 1791)

Figures 10A, B , 11

######### Specimens examined

(n=2). RMNH Coel 40506, East Kalimantan–Berau Expedition station BER.14, lighthouse northeast side of Pulau Panjang, Berau Islands, East Kalimantan (02°23'14"N, 118°12'34"E), depth = 12 m, collected on October 09, 2003 by B.W. Hoeksema; RMNH Coel 40509, East Kalimantan–Berau Expedition station BER.01, east side of Pulau Derawan, Berau Islands, East Kalimantan (02°17'32"N, 118°15'43"E), depth = 14 m, collected on October 11, 2003 by B.W. Hoeksema.

######### Photographic records

(n=7). west Pulau Barang Caddi, Spermonde Archipelago, South Sulawesi (05°05'08"S, 119°18'55"E), October 06, 1997; east Bone Lola shoal, Spermonde Archipelago, South Sulawesi (05°03'15"S, 119°21'30"E), October 27, 1997; east Pulau Kudingareng Keke, Spermonde Archipelago, South Sulawesi (05°06'15"S, 119°17'35"E), September 17, 1997; north Pulau Kudingareng Keke, Spermonde Archipelago, South Sulawesi (05°06'07"S, 119°17'15"E), October 1, 1997; station BER.01, east Pulau Derawan, East Kalimantan, Berau Islands (02°17'32"N, 118°15'43"E), October 11, 2003; station BER.14, lighthouse northeast Pulau Panjang Island, East Kalimantan, Berau Islands (02°23'14"N, 118°12'34"E), October 9, 2003; station BER.24, southeast Pulau Samama, East Kalimantan, Berau Islands (02°07'51"N, 118°20'23"E), October 15, 2003.

######### Description.

**Figure 10.**
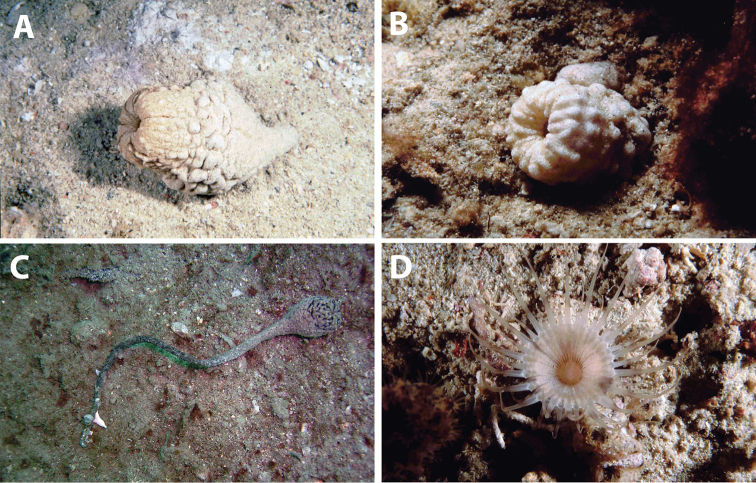
Images of *Sphenopus* species from photographic records in this study. **A** *Sphenopus marsupialus* at east Bone Lola shoal, Spermonde Archipelago, South Sulawesi, October 27, 1997 **B** *Sphenopus marsupialus* at station BER.14, lighthouse northeast Pulau Panjang Island, East Kalimantan, Berau Islands, October 9, 2003 **C** *Sphenopus pedunculatus* specimen RMNH Coel 40507, Kepulauan Seribu Expedition station SER.29, north side of Pulau Tikus, Thousand Islands off Jakarta, northwest Java, depth = 30 m, collected on September 18, 2005 by B.W. Hoeksema; and **D** *Sphenopus pedunculatus* specimen RMNH Coel 40510, East Kalimantan–Berau Expedition station BER.03, south side of Pulau Derawan, East Kalimantan, depth = 15 m, collected on October 21, 2003 by B.W. Hoeksema.

The type species of the azooxanthellate genus *Sphenopus*, this species has an Indo-West Pacific distribution (Reimer et al. 2012b). Uniquely for the order, species in this genus are unitary (not colonial), and usually free-living, as they are not attached to substrate, and instead embedded in sand or loose gravel/substrate (Figures 10A, B). Individuals can often grow to large sizes (for zoantharians); up to several cm in both length and polyp diameter. Taxonomic examination of this genus is quite limited, with only two recent studies (Soong et al. 1999, Reimer et al. 2012b), both of which clearly state that further research is needed to more clearly understand this group.

Specimen RMNH Coel 40506 consists of seven polyps, with an average height of 24.4 mm (range 18.5 to 30 mm), and an average width of 8.4 mm (range 6 to 11 mm). The non-peduncle portions of the polyps are 15–20 mm in height, with the remainder made up of peduncle.

Specimen RMNH Coel 40506 has some polyps (five of seven) somewhat different in morphology from RMNH Coel 40509 and other Naturalis *Sphenopus marsupialis* specimens from the Indian Ocean. These polyps have regularly spaced small round “tubercles” (approx. 1 mm in diameter) on the upper half of their scapus arranged in vertical lines (n=8–14 vertical tubercle lines on each polyp, with 6–10 tubercles per line), making this portion of the polyp appear furrowed. As well, polyps have a small, stubby “peduncle” (2 to 5 mm in width) that is not attached to any hard substrate, intermediate between *Sphenopus marsupialis* with its completely rounded bottom end and *Sphenopus pedunculatus* with its long, attached peduncle. For now, we identify these specimens as *Sphenopus marsupialis* as their peduncles were not attached to the substrate, but it is clear more examination of these specimens is needed.

Specimen RMNH Coel 40509 consists of two polyps of different sizes, with the smaller one being 16 by 5 mm, and the larger one 24 by 15 mm. Both polyps have no peduncle and are tapered. Both polyps are somewhat rugged on their outer surface, with no discernable tubercles, and have intermittent (=not one clear stripe) small darker vertical patterns in between the capitulary ridges only on the top 3–5 mm of the oral end of polyps.

######### Distribution.

**Figure 11.**
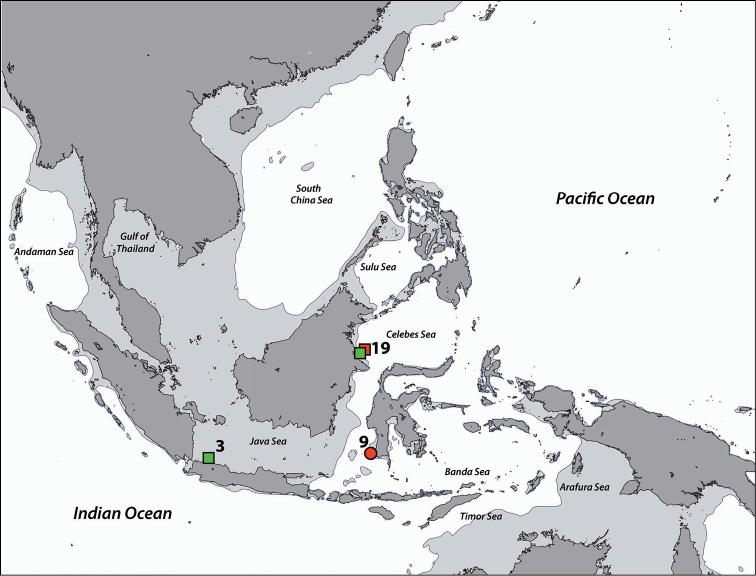
Distribution of *Sphenopus* species from specimens and photographic records from this study. *Sphenopus marsupialus* specimens in red, and *Sphenopus pedunculatus* in green. Region numbers correspond to locations given in species’ information. Boxes indicate presence of specimens (with or without photographic records), while circles indicate only photographic records. Overlapping symbols indicate the same region.

**Regions recorded in this study** (Figure 11). Spermonde Archipelago (9), Berau Islands (19).

**Previous records.** This species has been reported from many locations in the Indo-West Pacific, including Taiwan (Soong et al. 1999) and Brunei Darussalam (Reimer et al. 2012b).

######### Remarks.

Specimen RMNH Coel 40506 may be similar to a putative undescribed *Sphenopus* species mentioned in Soong et al. (1999) from Taiwan based on its smaller size.

######## 12. Sphenopus pedunculatus

Taxon classificationAnimaliaZoanthariaSphenopidae

Hertwig, 1888

Figures 10C, D , 11

######### Specimens examined

(n=2). RMNH Coel 40507, Kepulauan Seribu Expedition station SER.29, north side of Pulau Tikus, Thousand Islands off Jakarta, northwest Java (05°51'13"S, 106°34'43"E), depth = 30 m, collected on September 18, 2005 by B.W. Hoeksema; RMNH Coel 40510, East Kalimantan–Berau Expedition station BER.03, south side of Pulau Derawan, East Kalimantan (02°17'03"N, 118°14'49"E), depth = 15 m, collected on October 21, 2003 by B.W. Hoeksema.

######### Photographic records

(n=2). Images of RMNH Coel. 40507 and RMNH Coel 40510 as above.

######### Description.

This azooxanthellate species was originally described from the Philippines, and has not been reported in the literature for over 80 years, excepting two brief mentions in Reimer et al. (2012b). Easily discernable from other *Sphenopus* species by the presence of a ‘foot’ (=peduncle) that is attached to substrate (e.g. small rocks).

The two specimens here varied in length from 33 to 62 mm in polyp length, and had a width between 9 to 11 mm (polyp head). The “swollen”, non-peduncle part of the polyp was between 15 to 20 mm in height, with the remainder of the length made up of the peduncle, which was between 0.5 to 3 mm in width. RMNH Coel 40507 polyps were generally smooth in appearance, while the upper portions of polyps of RMNH Coel 40510 were somewhat rugged, with small round tubercules 0.5 mm in diameter roughly arranged in vertical lines. The spaces between these small tubercules were colored a much darker color than the remainder of the polyps’ outer surfaces. The peduncle of specimens and images (Figure 10C) are much thinner and longer than the sketch in Hertwig (1888). However, so few data are available for this (and other *Sphenopus* species) that currently nothing is known about intraspecific variation, and for now, we group these two specimens within this species.

######### Distribution.

**Regions recorded in this study** (Figure 11). Northwest Java (3), Berau Islands (19).

**Previous records.** This species was originally described from the Philippines, but has not been mentioned in recent literature (except for Reimer et al. 2012b), and hence very little is known on its distribution or ecology.

######### Remarks.

It is unknown as to whether the peduncle is a morphological characteristic that forms only when there is a hard substrate available, and this needs to be investigated to confirm this is truly a different species from *Sphenopus marsupialis*.

##### Suborder Macrocnemina Haddon & Shackleton, 1891a

###### Family Hydrozoanthidae Sinniger, Reimer & Pawlowski, 2010

####### Genus *Hydrozoanthus* Sinniger, Reimer & Pawlowski, 2010

######## 13. Hydrozoanthus gracilis

Taxon classificationAnimaliaZoanthariaHydrozoanthidae

(Lwowsky, 1913) sensu Di Camillo et al. (2010)

Figures 12A, B , 13

######### Specimens examined

(n=3). RMNH Coel 40692, Snellius–II Expedition station 4.098, East Komodo, Komodo (08°29'54"S, 119°38'06"E), depth = 75 m, collected on September 19, 1984 by rectangular dredge; RMNH Coel. 40518, Snellius–II Expedition station 4.022, north Pulau Mai, Maisel Islands, Banda Sea (05°29'S, 127°32'E), depth = 0 to 1.5 m, collected on September 7, 1984; RMNH Coel 3816, Snellius Expedition, Sipankat Island, near Siburu Island, Sulu Islands, Philippines, collected on September 10–14, 1929.

######### Photographic records

(n=5). Southwest Nusa Penida, eastern Bali (08°49'S, 115°34"E), May 25, 1998; Desa Ped, Nusa Penida, Lombok Strait, east Bali (08°40'28"S, 115°30'50"E), May 25, 1998; east Tanjung Taal, Nusa Lembongan, Lombok Strait, east Bali (08°39'33"S, 115°26'37"E), May 24, 1998; Fauna Malesiana Maluku Expedition station MAL.21, west of Lilibooi, north coast Ambon Bay, Ambon, Moluccas (03°44'S, 128°02'E), November 20, 1996; East Kalimantan Program station BER.16, northeast Pulau Maratua, East Kalimantan, Berau Islands (02°17'29"N, 118°35'29"E), October 10, 2003.

######### Description.

**Figure 12.**
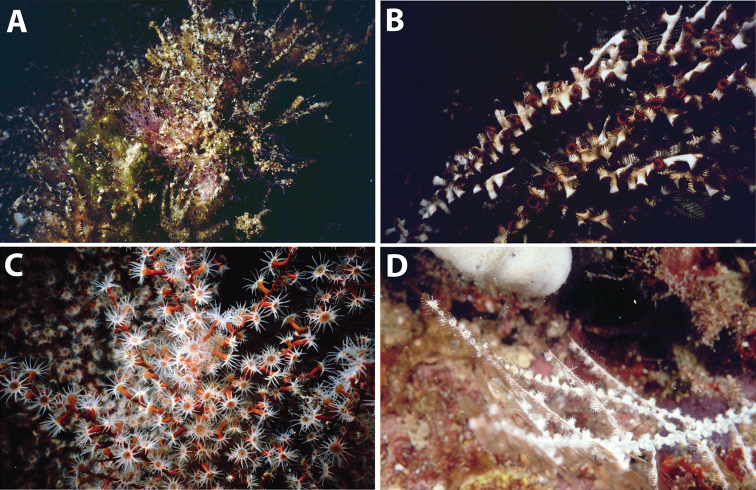
Images of *Hydrozoanthus* species from photographic records in this study. **A** *Hydrozoanthus gracilis* from Fauna Malesiana Maluku Expedition, station MAL.21, west of Lilibooi, north coast Ambon Bay, Ambon, Moluccas, November 20, 1996 **B** *Hydrozoanthus gracilis* at Southwest Nusa Penida, east Bali, May 25, 1998 **C** *Hydrozoanthus* sp. 1 at Balicasag Island, Cebu Strait, Philippines, November 21, 1999; and **D** *Hydrozoanthus* sp. 2 at East Kalimantan Program station BER.20, Tanjung Pandan shoal, Southwest of Pulau Panjang, East Kalimantan, Berau Islands, October 22, 2003.

As originally and previously described (Di Camillo et al. 2010), this azooxanthellate, colonial species is found as an epibiont on hydrozoans, particularly *Plumularia habereri* Stechow, 1909. In this study, this species consists of only one morphotype, with a gray to brown scapus, and reddish-brown oral disk and tentacles (Figure 12B). The appearance matches well with the morphotype of the species observed by Di Camillo et al. (2010).

In this study, measurements are only available for two specimens, with polyps averaging 2.4 mm in height and 2.1 mm in width. These data also fit well with Di Camillo et al. (2010), who mention polyp heights of 2–5 mm, widths of 1.6 to 3 mm, with 32 tentacles and mesenteries.

######### Distribution.

**Figure 13.**
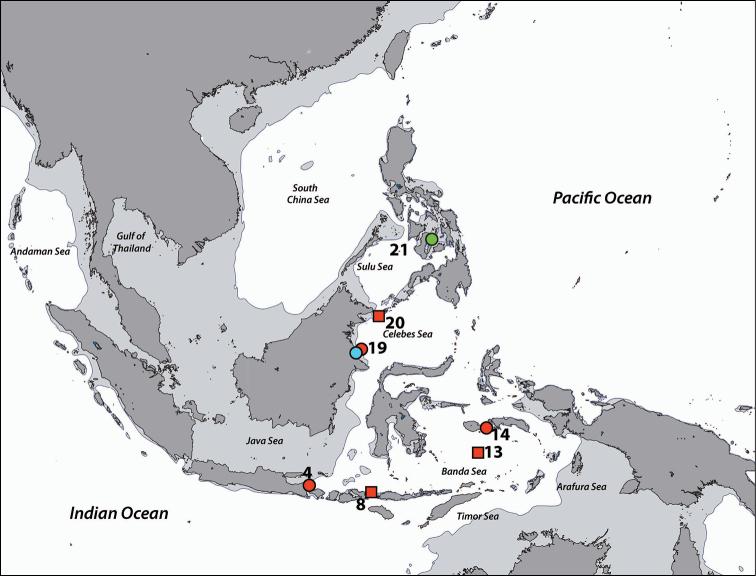
Distribution of *Hydrozoanthus* species from specimens and photographic records from this study. *Hydrozoanthus gracilis* specimens in red, *Hydrozoanthus* sp. 1 in green, and *Hydrozoanthus* sp. 2 in blue. Region numbers correspond to locations given in species’ information. Boxes indicate presence of specimens (with or without photographic records), while circles indicate only photographic records. Overlapping symbols indicate the same region.

**Regions recorded in this study** (Figure 13). East Bali (5), Komodo Island (8), Maisel Islands (13), Moluccas (14), Berau Islands (19), Sulu Islands (20).

**Previous records.** Originally reported from Sagami Bay, Japan (Lwowsky 1913), and subsequently reported from Taiwan (Reimer et al. 2011c), New Caledonia (Sinniger 2006), and Indonesia (Sinniger et al. 2005, Di Camillo et al. 2010). It appears this species has an Indo-West Pacific distribution.

######### Remarks.

This morphotype differs from the other known morphotype of the species (sensu Carlgren 1934) associated with this binomen, which is yellow in coloration. The original description of the species from Sagami Bay, Japan by Lwowsky (1913) was of a “gray, sandy” morphotype, but this was preserved in formalin, and thus could be either morphotype discussed here, or even a different one altogether. Phylogenetic analyses have shown subtle differences of sequences of specimens within this species (Sinniger et al. 2008), indicating that taxonomic revision may be needed in the future for this species group.

######## 14. Hydrozoanthus sp. 1

Taxon classificationAnimaliaZoanthariaHydrozoanthidae

Figures 12C , 13

######### Specimens examined.

NA.

######### Photographic records

(n=1). Balicasag Island, Cebu Strait, Philippines (09°31'01"N 123°41'04”E), November 21, 1999.

######### Description.

Similar to *Hydrozoanthus gracilis* above, this azooxanthellate, colonial species is found as an epibiont on *Plumularia habereri*. As described in Di Camillo et al. (2010; as *Parazoanthus* sp.), this species has much smaller polyps than *Hydrozoanthus gracilis*, forming colonies only on the main branch(es) of *Plumularia habereri* colonies. Polyps are much less incrusted than *Hydrozoanthus gracilis*. The *Plumularia habereri* colonies hosting this species are much bigger than those with *Hydrozoanthus gracilis*, as shown by (Di Camillo et al. 2010). Red scapus with yellow tentacles, 22 to 24 tentacles slightly longer than oral disk diameter (Figure 12C).

######### Distribution.

**Regions recorded in this study** (Figure 13). Cebu (21).

**Previous records.** Reported from Bunaken, North Sulawesi, in Di Camillo et al. (2010).

######### Remarks.

This undescribed species was informally and well described by Di Camillo et al. (2010) as “*Parazoanthus* sp.”. Specimens and DNA sequences are needed to properly describe this species.

######## 15. Hydrozoanthus sp. 2

Taxon classificationAnimaliaZoanthariaHydrozoanthidae

Figures 12D , 13

######### Specimens examined.

NA.

######### Photographic records

(n=1). East Kalimantan Program station BER.20, Tanjung Pandan shoal, southwest of Pulau Panjang, East Kalimantan, Berau Islands (02°19'15"N, 118°06'33"E), October 22, 2003.

######### Description.

Similar to *Hydrozoanthus gracilis* and *Hydrozoanthus* sp. 1 above, this azooxanthellate, colonial species is found as an epibiont on *Plumularia habereri*. Similar to *Hydrozoanthus* sp. 1, this completely white species has much smaller polyps than *Hydrozoanthus gracilis*, forming colonies only on the main branch(es) of *Plumularia habereri* colonies (Figure 12D). Polyps are much less incrusted than *Hydrozoanthus gracilis*.

######### Regions recorded in this study

(Figure 13). Berau Islands (19).

**Previous records.** NA.

######### Remarks.

This undescribed species may be a different colored morphotype of *Hydrozoanthus* sp. 1 (above) informally described by Di Camillo et al. (2010) as “*Parazoanthus* sp.”. Specimens and DNA sequences are needed to properly describe this species.

####### Genus *Terrazoanthus* Reimer & Fujii, 2010

######## 16. Terrazoanthus sp. 1

Taxon classificationAnimaliaZoanthariaHydrozoanthidae

Figures 14A, B , 15

######### Specimens examined

(n=1). RMNH Coel 40469, Fauna Malesiana Maluku Expedition station MAL.05, Leitimur, outer Ambon Bay, Tanjung Bentang, Ambon, Moluccas (03°35'S, 128°05'E), depth = NA, collected on November 7, 1996 by J.C. den Hartog.

######### Photographic records

(n=1). West Pulau Badi, Spermonde Archipelago, South Sulawesi (04°58'06"S, 119°16'57"E), September 29, 1997.

######### Description.

**Figure 14.**
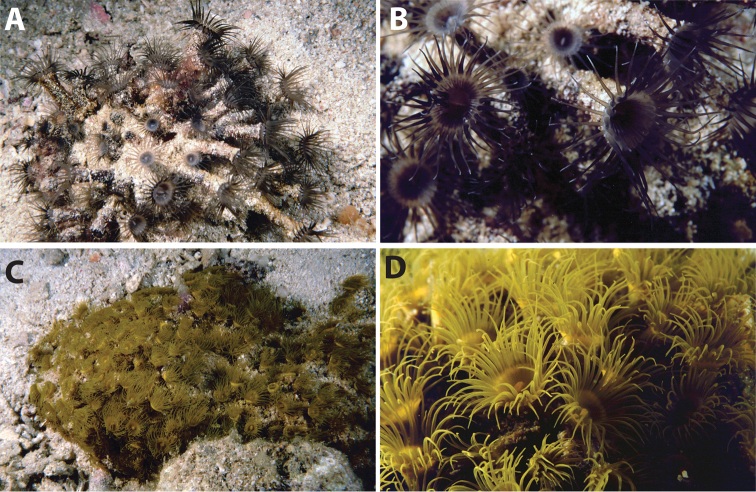
Images of *Terrazoanthus* species from photographic records in this study. **A** and **B** *Terrazoanthus* sp. 1 at the west side of Pulau Badi, Spermonde Archipelago, South Sulawesi, September 29, 1997; and **C** and **D** *Terrazoanthus* sp. 2 at the west side of Bone Lola shoal, Spermonde Archipelago, South Sulawesi, April 22, 1998.

Azooxanthellate. Polyps well free and clear of coenenchyme. Outer surface of polyps covered with dense incrustation of irregularly sized sand particles, reminiscent of *Microzoanthus* sp. Oral disk semi-translucent with dark, almost black coloration, except for oral opening, which is much lighter in color. 40 to 50 tentacles, at least as long as oral disk diameter, with same blackish coloration as oral disk, with terminal 1/4 whitish in coloration. Colonies attached to non-living substrate. Specimen RMNH Coel 40469 is apparently a fragment of a whole colony, while the photographic record shows a colony of approximately 50 polyps arising from a common coenenchyme (Figure 14A). The single specimen had polyps averaging 6.8 mm in length (n=3) and 3.1 mm in width (n=6).

######### Distribution.

**Figure 15.**
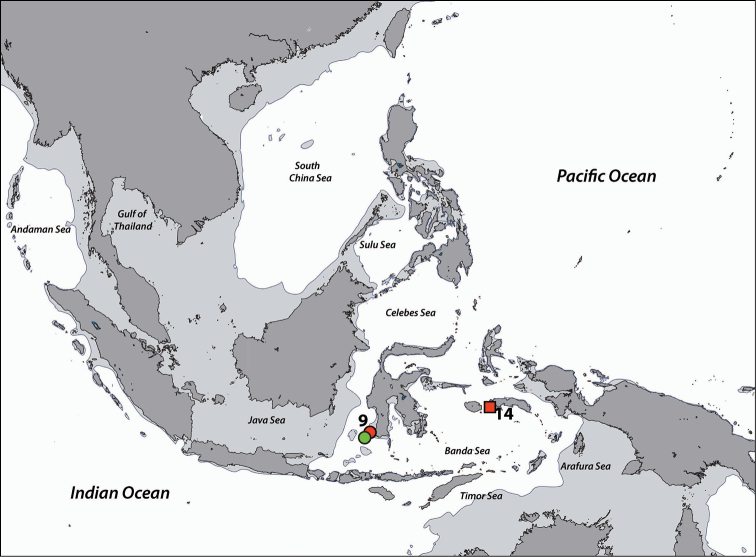
Distribution of *Terrazoanthus* species from specimens and photographic records from this study. *Terrazoanthus* sp. 1 specimens in red, and *Terrazoanthus* sp. 2 in green. Region numbers correspond to locations given in species’ information. Boxes indicate presence of specimens (with or without photographic records), while circles indicate only photographic records. Overlapping symbols indicate the same region.

**Regions recorded in this study** (Figure 15). Spermonde Archipelago (9), Moluccas (14).

**Previous records.** None, although similar undescribed specimens have been photographed in the Philippines (P. Poppe, pers. comm.), and collected from Okinawa, Japan (Reimer, unpubl. data), indicating a potential West Pacific distribution.

######### Remarks.

This species is similar in appearance but different in coloration to *Terrazoanthus onoi* from the Galapagos and west coast of Central and South America.

######## 17. Terrazoanthus sp. 2

Taxon classificationAnimaliaZoanthariaHydrozoanthidae

Figures 14C, D , 15

######### Specimens examined.

NA.

######### Photographic records

(n=1). West Bone Lola shoal, Spermonde Archipelago, South Sulawesi (05°03'15"S, 119°21'15"E), April 22, 1998.

######### Description.

With only a single photographic record available, even an informal description of this undescribed species is limited. Asides from yellow coloration, this species is outwardly similar to *Terrazoanthus* sp. 1 above. Polyps appear to be more crowded than in *Terrazoanthus* sp. 1, with 40 to 54 yellow tentacles longer than oral disk diameter (Figure 14D).

######### Regions recorded in this study

(Figure 15). Spermonde Archipelago (9).

######### Overall distribution.

Unknown, although similar specimens have been photographed in the Philippines (P. Poppe, pers. comm.).

######### Remarks.

This species has been traded in the aquarium trade as “yellow polyps”, and is thought to be distributed primarily in Indonesia, yet no museum specimens exist, preventing this species from being formally described. Colonies often appear to be intermixed with *Zoanthus* spp. colonies in shallow water (J.D. Reimer, pers. obs.). Although undescribed, this putative species has been placed with the genus *Terrazoanthus* based on DNA sequences acquired from aquarium trade polyps (Sinniger et al. 2005, Reimer and Fujii 2010).

###### Family Parazoanthidae Delage & Hérouard, 1901

####### 18. Parazoanthidae sp. 1

Taxon classificationAnimaliaZoanthariaParazoanthidae

Figures 16A, B , 17

######## Specimens examined

(n=2). RMNH Coel 40766, Fauna Malesiana Maluku Expedition station MAL.09, southwest coast, Ambon, Latuhalat, Moluccas (03°46'S, 128°06'E), depth = to 24 m, collected on November 11, 1996; RMNH Coel 40768, Snellius Expedition, Pulau Bo Islands, Halmahera Sea, collected on October 5, 1930.

######## Photographic records

(n=1). Station BER.30, north of Lighthouse 1 Reef, south of Pulau Derawan, East Kalimantan, Berau Islands (02°16'02"N, 118°14'23"E), October 22, 2003.

######## Description.

**Figure 16.**
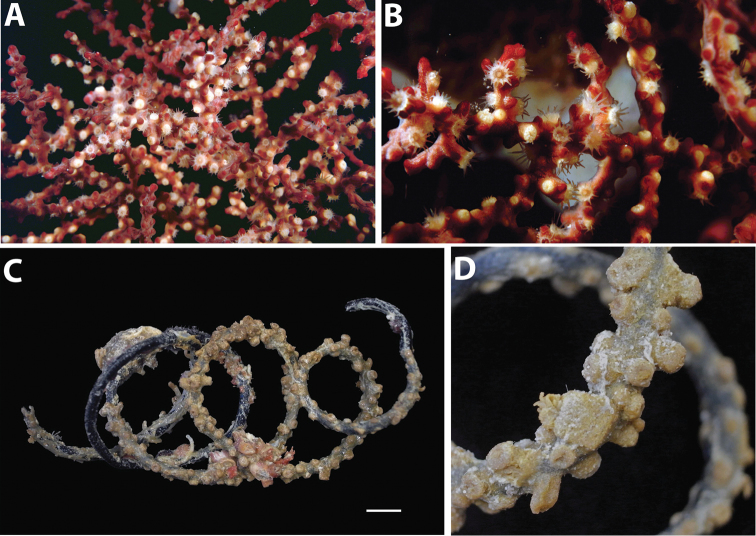
Images of Parazoanthidae sp. 1 and Parazoanthidae sp. 2 from specimens and photographic records in this study. **A** and **B** Parazoanthidae sp. 1 at station BER.30, north of Lighthouse 1 Reef, south of Pulau Derawan, East Kalimantan, Berau Islands, October 22, 2003. Note octocoral polyps on antipatharian on left side of image **C** and **D** Parazoanthidae sp. 2 specimen RMNH Coel 40762, Snellius–II Expedition, Station 4.227, west Pulau Tinanja, Taka Bone Rate, depth = 60 m, collected on October 15, 1984 by rectangular dredge. Scale in **C** 1 cm.

Azooxanthellate, epibiotic on *Keroeides* sp., polyps approximately the same height as width (approximately 1–3 mm), connected by coenenchyme visible on the outer surface of the octocoral colony. Polyps numerous, placed between smaller octocoral polyps, pale yellow in coloration, with outer surface of polyps slightly reddish in color similar to host octocoral. Tentacles relatively short, approximately half of the oral disk diameter, also pale yellow, and approximately 20 in number (Figure 16A).

Specimen RMNH Coel 40766 is larger than RMNH Coel 40768 (polyp average width 2.6 mm vs 1.6 mm, respectively). However, the latter specimen is quite old (from the original Snellius Expedition) and this difference may be due to fixation methods.

######## Distribution.

**Figure 17.**
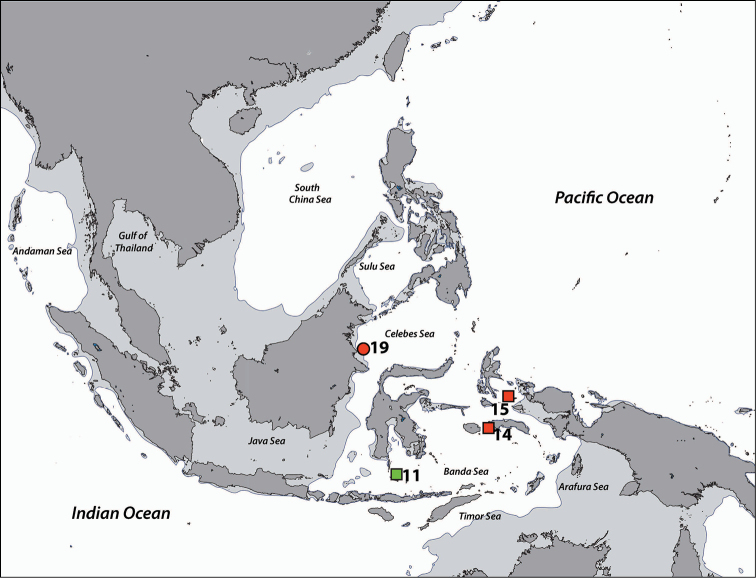
Distribution of Parazoanthidae sp. 1 and Parazoanthidae sp. 2 from specimens and photographic records from this study. Parazoanthidae sp. 1 specimens in red, and Parazoanthidae sp. 2 in green. Region numbers correspond to locations given in species’ information. Boxes indicate presence of specimens (with or without photographic records), while circles indicate only photographic records.

**Regions recorded in this study** (Figure 17). Moluccas (14), Bo Islands (15), Berau Islands (19).

**Previous records.** NA.

######## Remarks.

Only two specimens and one photographic record of this undescribed species exist. However, these records are each from different expeditions, and it is reasonable to expect that this species is at least distributed in the Banda and Celebes Seas.

Recently, many different genera in the family Parazoanthidae have been described based on a combination of epizoitic relationships and phylogenetic analyses (e.g. Sinniger et al. 2010, 2013). However, no parazoanthids have been reported in association with *Keroeides*, and therefore currently it is impossible to place these specimens and records into a genus without both further examination of specimens combined with DNA sequence data.

####### 19. Parazoanthidae sp. 2

Taxon classificationAnimaliaZoanthariaParazoanthidae

Figures 16C, D , 17

######## Specimens examined

(n=1). RMNH Coel 40762, Snellius–II Expedition, Station 4.227, west Pulau Tinanja, Taka Bone Rate (06°32'48"S, 121°09'36"E), depth = 60 m, collected on October 15, 1984 by rectangular dredge.

######## Photographic records.

NA.

######## Description.

Epibiotic on *Cirripathes* sp. (specimen RMNH Coel 24832). Polyps of this azooxanthellate zoantharian specimen are relatively small (average width 2.1 mm, n=8 polyps) and do not protrude much from the coenenchyme, with polyp height approximately same as width. Polyps and coenenchyme are heavily encrusted, and golden yellow-brown in color. Coenenchyme forms a thin sheath over the antipatharian surface. Capitulary ridges not clearly discernable. Polyps form semi-regular vertical rows over short distances of the antipatharian (e.g. approx. 5 cm), but with no observable pattern for the entire colony (Figure 16C). Colony encrusts the top approximately 1/2 of the *Cirripathes* specimen; starting approximately 15 cm from the bottom holdfast. The *Cirripathes* colony’s proximal tip appears to be broken off and missing.

######## Distribution.

**Regions recorded in this study** (Figure 17). Taka Bone Rate (11).

**Past records.** NA.

######## Remarks.

This species may belong to genus *Antipathozoanthus*, which was described recently by Sinniger et al. (2010) and includes species from both the Atlantic and Indo-Pacific, with reports of specimens also from the Red Sea (Reimer et al. 2014b). It is likely several undescribed *Antipathozoanthus* species are present in the Indo-Pacific, as only one *Antipathozoanthus* species from the Galapagos has been formally described. In situ images and DNA sequences are needed to formally describe this species.

####### Genus *Parazoanthus* Haddon & Shackleton, 1891a

######## 20. Parazoanthus sp. 1

Taxon classificationAnimaliaZoanthariaParazoanthidae

Figures 18A , 19

######### Specimens examined.

NA.

######### Photographic records

(n=3). West side of Pulau Kudengareng Keke, Spermonde Archipelago, South Salawesi (05°06'20"S, 119°17'03"E), June 4, 1997; Cabilao Island, Cebu Strait, Philippines (09°52'35”N, 123°46'33”E), November 16, 1999; station WAK.24, Ndaa Atoll northwest outer slope, REA Wakatobi National Park, Tukang Besi Islands, Wakatobi, Southeast Sulawesi, (05°38'46"S, 124°02'42"E), May 12, 2003.

######### Description.

**Figure 18.**
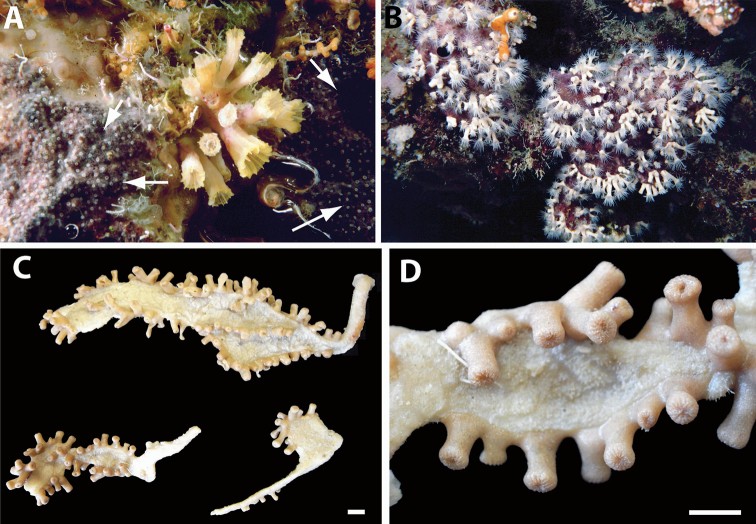
Images of *Parazoanthus* species from specimens and photographic records in this study. **A** *Parazoanthus* sp. 1 on cave ceiling at station WAK.24, Ndaa Atoll northwest outer slope, REA Wakatobi National Park, Southeast Sulawesi, Tukang Besi Islands, Wakatobi, May 12, 2003 **B** *Parazoanthus* sp. 2 at Southeast Likuan, Bunaken, North Sulawesi, May 10, 1998; and **C** and **D** *Parazoanthus* sp. 3 specimen RMNH Coel 40545, Snellius–II Expedition station 4.051, east of Melolo, northeast Sumba, depth = 75 to 90 m, collected on September 13, 1984 by rectangular dredge. Scales in **C** and **D** 1 cm.

Very small (polyp diameter likely approximately 1 mm) azooxanthellate polyps regularly spaced and embedded within encrusting sponge tissue (Figure 18A). Polyps differentially colored from sponges; dark red (Cebu), yellow (Pulau Kudengareng Keke), white (Wakatobi). Tentacles up to 24 in number (in images here), as long as diameter of oral disk.

######### Distribution.

**Figure 19.**
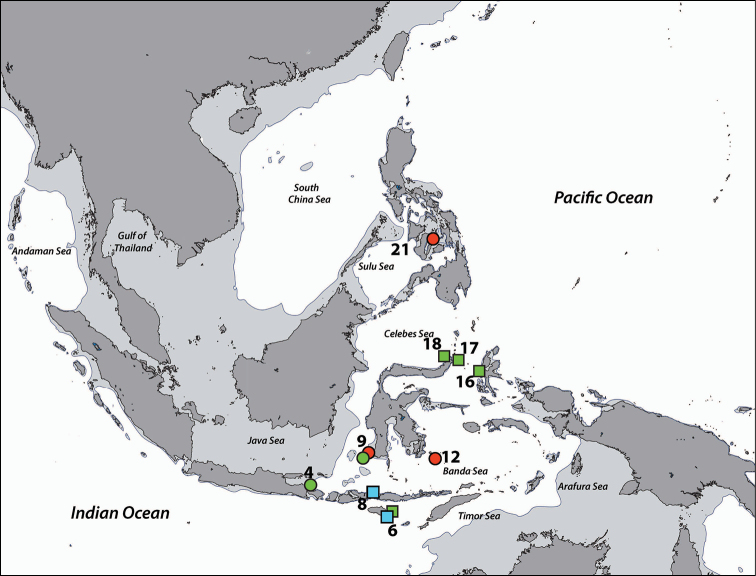
Distribution of *Parazoanthus* species from specimens and photographic records from this study. *Parazoanthus* sp. 1 specimens in red, *Parazoanthus* sp. 2 in green, and *Parazoanthus* sp. 3 in blue. Region numbers correspond to locations given in species’ information. Boxes indicate presence of specimens (with or without photographic records), while circles indicate only photographic records. Overlapping symbols indicate the same region.

**Regions recorded in this study** (Figure 19). Spermonde Archipelago (9), Tukang Besi Islands (12), Cebu (21).

**Past records.** Previously, similar specimens have been reported from Japan (Sinniger et al. 2008) and the Red Sea (Reimer et al. 2014b).

######### Remarks.

Based on phylogenetic data (J. Montenegro, F. Sinniger and J.D. Reimer, unpubl. data) it appears that this group includes several undescribed species. The species has been found on cave ceilings (Figure 18A), which may explain why it is azooxanthellate as in some other hexacorals with white polyps (Hoeksema 2012b, Reimer et al. 2014, Irei et al. subm).

######## 21. Parazoanthus sp. 2

Taxon classificationAnimaliaZoanthariaParazoanthidae

Figures 18B , 19

######### Specimens examined

(n=4). RMNH Coel 40544, Snellius–II Expedition Station 4.061, east of Melolo, northeast Sumba (09°54'12"S, 120°43'30"E), depth = 50 m, collected on September 15, 1984 by rectangular dredge; RMNH Coel. 40570, station 9, reef slope of southwest Pulau Nain, Bunaken, North Sulawesi (01°46'N, 124°45'E), collected on May 8, 1998 by B.W. Hoeksema; RMNH Coel 40572, Ternate Expedition Station TER.27, Tanjung Ratemu (south of river), west Halmahera Sea, North Moluccas (00°54'45"N, 127°29'10"E), depth = 20 m, collected on November 8, 2009 by B.W. Hoeksema; RMNH Coel 40757, Indonesia 2012 Expedition, Station LEM.34, west Pulau Sarena Kecil Lembeh, North Sulawesi (01°27'26"N, 125°13'31"E), depth = 22 m, collected on February 17, 2012 by B.W. Hoeksema.

######### Photographic records

(n=3). West Pulau Kudingareng Keke, Spermonde Archipelago, South Sulawesi (05°06'20"S, 119°17'03"E), June 4, 1997; southeast Likuan, Bunaken, North Sulawesi (01°36'N, 124°47'E), May 10, 1998; Main coast, West Bali (08°06'50"S, 114°30'40"E), May 22, 1998.

######### Description.

Azooxanthellate, epibiotic on encrusting sponges, with 3 to 6 polyps arising in groups from a common coenenchyme, or occasionally arising in rows from stolons (Figure 18B). Polyps (oral disk, tentacles, scapus) pale yellow/cream in color. 36 to 40 tentacles, longer in length than oral disk diameter. Specimens’ preserved polyps (n=2 specimens, 10 polyps per specimen) averaged 5.8 mm in height (range 4 to 8 mm) and 3.3 mm in width (range 2.5 to 5 mm).

######### Distribution.

**Regions recorded in this study** (Figure 19). West Bali (4), northeast Sumba (6), Spermonde Archipelago (9), west Halmahera Sea (16), Lembah Strait (17), Bunaken (18).

**Past records.** NA.

######### Remarks.

The only sponge-associated *Parazoanthus* species formally described from the Indo-Pacific are *Parazoanthus elongatus* McMurrich, 1904 from the west coast of South America and New Zealand (Sinniger and Haussermann 2009) and *Parazoanthus darwini* Reimer & Fujii, 2010 from the Galapagos. Thus, no similar species have been reported from past or recent zoantharian work in surrounding CIP regions, and therefore it is likely that these specimens constitute an undescribed species. Although *Parazoanthus* has recently been taxonomically redescribed (Sinniger et al. 2010), and the species now only encompasses sponge-associated species, the genus is still paraphyletic and taxonomic revision is needed. DNA sequences from undescribed species are needed to correctly place specimens such as these into the correct clade.

######## 22. Parazoanthus sp. 3

Taxon classificationAnimaliaZoanthariaParazoanthidae

Figures 18C, D , 19

######### Specimens examined

(n=2). RMNH Coel 40525, Snellius–II Expedition station 4.100, east of Komodo Island (08°28.6'S, 119°37.3'E), depth 91 m, collected on September 19, 1984 by rectangular dredge; RMNH Coel 40545, Snellius–II Expedition station 4.051, east of Melolo, northeast Sumba (09°53.5'S, 120°42.7'E), depth 75-90 m, collected on September 13, 1984 by rectangular dredge.

######### Photographic records.

NA.

######### Description.

This putative azooxanthellate species is similar in size to *Parazoanthus* sp. 2 above, with polyps of average 6.1 mm in height (range 2.5 to 10 mm; n=2 colonies) and average width of 3.2 mm (range 2 to 4 mm). Some small dark incrustations visible on lower half (=aboral) of polyps’ scapus. Approximately 20 capitulary ridges, indicating tentacle counts of approximately 40. Polyps range from cream (RMNH Coel 40525) to tan (RMNH Coel 50545) in color when preserved. Polyps arise from a well-developed stoloniferous coenenchyme in rows, with most found along the upper and outer edges of flat, paddle-shaped sponges (Figures 18C, D). No polyps found on the lower ‘foot’ or ‘stalk’ of sponges.

######### Distribution.

**Regions recorded in this study** (Figure 19). Northeast Sumba (6), Komodo Island (8).

**Past records.** NA.

######### Remarks.

Similar in size to *Parazoanthus* sp. 2 above, we have included these two specimens as a separate putative species in this study. This is partly due to specimens being in association with a different sponge species (compare Figures 18B, C), as host specificity may differ between species (e.g. Crocker and Reiswig 1981; Swain and Wulff 2007). As well, *Parazoanthus* sp. 3 specimens are from deeper depths (75 to 91 m as opposed to 20 to 50 m) than *Parazoanthus* sp. 2.

###### Family Epizoanthidae Delage & Hérouard, 1901

####### Genus *Epizoanthus* Gray, 1867

######## 23. Epizoanthus illoricatus

Taxon classificationAnimaliaZoanthariaEpizoanthidae

Tischbierek, 1930

Figures 20A , 21

######### Specimens examined

(n=4). RMNH Coel 40533, Snellius–II Expedition Station 4.222, south of Pulau Tarupa Kecil, Taka Bone Rate (06°31'30"S, 121°08'00"E), depth 58 m, collected on October 15, 1984 by rectangular dredge; RMNH Coel 40546, Snellius–II Expedition Station 4.051, east of Melolo, northeast Sumba (09°53'30"S, 120°42'42"E), depth = 75 to 90 m, collected on September 13, 1984 by rectangular dredge; RMNH Coel 40571, Ternate Expedition Station TER.27, Tanjung Ratemu, south of river, west Halmahera Sea (00°54'44"N, 127°29'10"E), depth = 20 m, collected on November 08, 2007 by B.W. Hoeksema; RMNH Coel 40758, station LEM.32, north Sarena Kecil, Lembeh Strait, North Sulawesi (01°27'26"N, 125°13'38"E), depth = 30 m, collected on February 16, 2012 by B.W. Hoeksema.

######### Photographic records

(n=6). West Menjangan, West Bali (08°05'33"S, 114°29'47"E) May 22, 1998 (3 different specimens); Maluku Expedition station MAL.21, north coast Ambon Bay, Tanjung Hatupero, east of Lilibooi, Ambon (03°44'S, 128°02'E), November 20, 1996; southeast Likuan, Bunaken, North Sulawesi (01°36'N, 124°47'E), May 10, 1998; station BER.04, south Pulau Derawan, East Kalimantan (02°17'03"N, 118°14'49”E), October 18, 2003.

######### Description.

**Figure 20.**
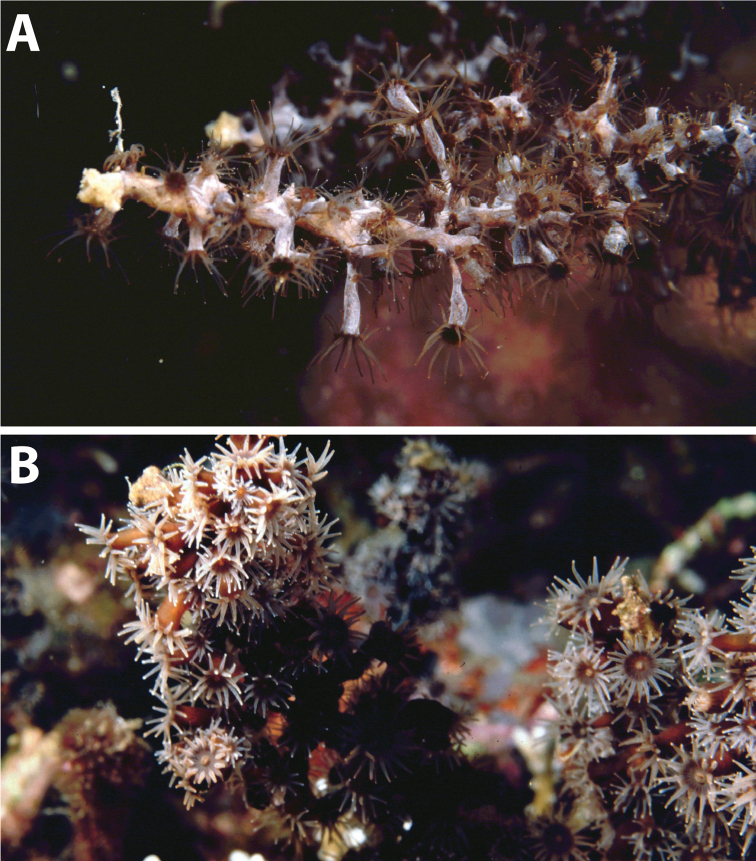
Images of *Epizoanthus* species from specimens and photographic records in this study. **A** *Epizoanthus illoricatus* at west Pulau Menjangan, West Bali, May 22, 1998; and **B** Epizoanthus aff. illoricatus at Tulamben, east coast of Bali, July 10, 1997.

Originally described from the Philippines. Azooxanthellate. Polyps of this species often grow at the outer bends of the zig-zag shaped tubes, and combined with polyps’ smaller size and thin coenenchyme (Figure 20A), colonies appear to be much less crowded than as seen in Epizoanthus aff. illoricatus (Figure 20B).

Polyps of specimens in the RMNH collection are generally less than 1 mm in diameter, and never more than 2 mm, and of approximately equal height. Coenenchyme generally light gray in color, oral disk and tentacles semi-translucent brown. Tentacles in images 20–22 in number, much thinner than as seen in Epizoanthus aff. illoricatus below, with orange or white colored proximal tips, longer in length than oral disk diameter. The two deeper specimens (RMNH Coel 40533 and 40546) have highly developed thin coenenchymes covering the entire worm tubes’ surface, and are both dark black in color. On the other hand, the shallower specimens had some unitary polyps, and colonial polyps were often in clusters of two or three with poorly developed coenenchyme.

The morphological characters and dimensions observed in the specimens in this study agree well with the original description by Tischbierek (1930).

######### Distribution.

**Figure 21.**
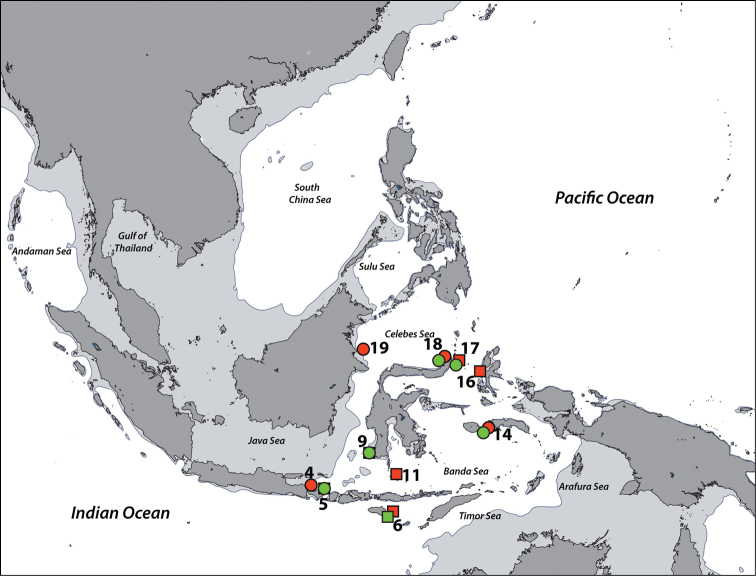
Distribution of *Epizoanthus* species from specimens and photographic records from this study. *Epizoanthus illoricatus* specimens in red, and Epizoanthus aff. illoricatus in green. Region numbers correspond to locations given in species’ information. Boxes indicate presence of specimens (with or without photographic records), while circles indicate only photographic records.

**Regions recorded in this study** (Figure 21). West Bali (4), northeast Sumba (6), Take Bone Rate (11), Moluccas (14), west Halmahera Sea (16), Lembeh Strait (17), Bunaken (18), Berau Islands (19).

**Past records.** Originally described from Manila, and subsequently reported from Taiwan (Reimer et al. 2013a), New Caledonia (Sinniger 2006), Palau (Reimer et al. 2014a), and Osprey Reef, Australia in the Coral Sea (Lindsay et al. 2012), indicating a western Indo-Pacific distribution. Found from specimens in this study as shallow as 20 m, and as deep as 90 m, similar to depths reported at Osprey Reef (=82.5 m) (Lindsay et al. 2012).

######### Remarks.

Until this report, any *Epizoanthus* spp. on a zig-zag shaped eunicid worm was recorded as *Epizoanthus illoricatus*. However, from the preliminary analyses here, it appears that there may be two or more species within this group. Thus, previous records must be treated with caution.

######## 24. Epizoanthus aff. illoricatus

Taxon classificationAnimaliaZoanthariaEpizoanthidae

Tischbierek, 1930

Figures 20B , 21

######### Specimens

(n=2). RMNH Coel 40536, Snellius–II Station 4.058, east of Melolo, northeast Sumba (09°51'S, 120°45'E), depth = 180 m, collected on September 14, 1984 by rectangular dredge; RMNH Coel 40547, Snellius–II Station 4.051, east of Melolo, northeast Sumba (09°53'30"S, 120°42'42"E), depth = 75 to 90 m, collected on September 13, 1984 by rectangular dredge.

######### Photographic records

(n=12). Desa Ped, north Nusa Penida, east Bali (08°40'28"S, 115°30'50"E), May 28, 1998; 4 specimens from Tulamben, east Bali (08°16'26"S, 115°35'28"E), July 9–10, 1997; Nusa Penida, east Bali, (08°40'23"S, 115°29'13"E), May 27, 1998; Kapoposang, Spermonde Archipelago, South Sulawesi (04°41'40"S, 118°54'55"E), June 24, 1997, August 8, 1997; west Pulau Samalona, Spermonde Archipelago, South Sulawesi (05°07'30"S, 119°20'15"E), September 16, 1997; Fauna Malesiana Maluku Expedition station MAL.10, south coast of Ambon Bay, east of Eri, Ambon (03°45'S, 128°08'E), November 12, 1996; Maluku Expedition station MAL.12, north coast near Morela, Ambon (03°33'S, 128°12'E), November 13–14, 1996; Maluku Expedition station MAL.19, Tanjung Batu Dua, east of Hatu, north coast Ambon Bay, Ambon (03°43'S, 128°03'E), November 19, 1996; Fauna Malesiana Marine Sulawesi Expedition station SUL.16, bay east of Tanjung Labuhankompeni, Pulau Lembeh, Lembeh Strait, North Sulawesi (01°26'N, 125°11'E), October 23, 1994; west Pulau Siladen, Bunaken, North Sulawesi (01°38'N, 124°46'E), May 2, 1998.

######### Description.

Azooxanthellate. As *Epizoanthus illoricatus* above, obligate epibiont on eunicid worms. Polyps of this putative species are at least twice as big in diameter as *Epizoanthus illoricatus* (average 2.1 mm, compared with a maximum of 2 mm for *Epizoanthus illoricatus*), and many times bigger in terms of volume. Additionally, both specimens have brown coenenchyme and scapus, different from the light gray coenenchyme and brownish oral disk reported for *Epizoanthus illoricatus* (Figure 20B). In situ images show colonies with cream, brown, red-brown, orange-brown or tan coenenchyme and scapus, often with white tentacles that are comparatively shorter and thicker than in *Epizoanthus illoricatus*. The coenenchyme of this putative species is much more developed than *Epizoanthus illoricatus*, with polyps arising from not only bends of the zig-zag shaped eunicid tube, but also from other locations. The result is a colony that has a higher density of polyps than *Epizoanthus illoricatus*. In *Epizoanthus illoricatus*, often the zig-zag shape of the eunicid tube is visible between polyps, but this is rarely the case in Epizoanthus aff. illoricatus (Figure 20B).

######### Distribution.

**Regions recorded in this study** (Figure 21): East Bali (5), northeast Sumba (6), Spermonde Archipelago (9), Moluccas (14), Lembeh Strait (17), Bunaken (18).

**Past records.** NA.

######### Remarks.

Although the two specimens here were found at deeper depths (75 to 190 m), numerous photographic records show that this species and *Epizoanthus illoricatus* have an overlapping depth range. Examination of DNA sequences combined with detailed morphological analyses should help clear up the relationship between this putative species and *Epizoanthus illoricatus*, although preliminary molecular analyses show differences between the two groups (H. Kise and J.D. Reimer, unpubl. data). It is likely records and museum specimens identified as *Epizoanthus illoricatus* from the central Indo-Pacific include both types mentioned in this study.

## Discussion

Examination of the Naturalis zoantharian collection resulted in 24 species being identified, 12 from suborder Brachycnemina and 12 from Macrocnemina. While by no means an extensive collection, with most specimens from Indonesia, these results indicate that the Central Indo-Pacific waters are at least as diverse in numbers of species, genera, and families as surrounding regions of Australia, Singapore, and Japan. In Australia, an examination of the brachycnemic shallow water zoantharians of the Great Barrier Reef indicated the presence of eight species (Burnett et al. 1997), while in Okinawa, 12 brachycnemic species have been previously reported (Reimer 2010), and in Taiwan 10 species (Reimer et al. 2013a). These previous reports did not include macrocnemic species, but from the brachycnemic results alone, Indonesia does appear to have zoantharian species diversity at least as high as Okinawa, one of the most well examined regions. Finally, as many macrocnemic species live in deeper areas or in caves and other less-examined ecosystems (Sinniger et al. 2013), we expect the number of zoantharian species in the shallow waters of Indonesia to be higher than the initial estimate in this study, and further investigations should confirm this idea.

The discussion of total numbers of shallow water zoantharian species is clearly still in the preliminary stages given the lack of focused sampling throughout most regions of the world, as well as the continuous discovery of new species and genera (Reimer et al. 2007a, Sinniger et al. 2010, Fujii and Reimer 2011, 2013). Still, the initial species numbers from this study should provide a basis upon which future zoantharian studies can build on. Furthermore, it should not be forgotten that the previous reports listed above from other Indo-Pacific regions were all conducted by zoantharian specialists collecting specimens in the field, while the Indonesian specimens in the Naturalis collection constitute only part of a broad sampling effort of benthic invertebrates without the participation of any zoantharian specialists. Thus, our prediction that the total number of shallow water zoantharian species in Indonesia will be considerably higher than reported in this study is almost certainly accurate, particularly given the recent discovery of widespread yet cryptic zoantharian species from coral reef environments (Fujii and Reimer 2011, 2013) not yet reported from Indonesia.

Further supporting the possibility of Indonesia harboring a diverse zoantharian fauna is the fact that the specimens examined in the Naturalis collection are primarily from eastern Indonesia (e.g. Sulawesi and Banda Sea, Fig. 1), with few or no specimens from other regions such as the islands of Java and New Guinea, and only one locality in the Philippines and Papua New Guinea. Future collection efforts must be focused on these unexamined regions if we are to obtain a clear understanding of zoantharian diversity in the CIP. Additionally, the deep sea (>200 m depth) has been recently speculated to harbor much undiscovered zoantharian diversity (Sinniger et al. 2013) and yet in this study only three of the zoantharian species were found in waters >50 m in depth. Exploring the deeper waters in the Indonesian region will undoubtedly result in further discoveries.

Of the 24 total species listed in this study, at least nine (and perhaps up to 12 if *Palythoa* spp. are included) are likely undescribed species. Some, such as *Terrazoanthus* sp. 2, have been known for years in the global aquarium trade, yet still no museum specimens exist, and thus we cannot formally describe them within this manuscript. Without formal descriptions and a clear understanding of species, future conservation work cannot proceed effectively, and immediate taxonomic efforts should focus on the obtaining of specimens and a formal description of this species. Similarly, many photographic records exist for *Neozoanthus* sp., yet no specimens are in the Naturalis collection.

Three other species that are almost certainly undescribed species are *Parazoanthus* sp. 1, *Parazoanthus* sp. 2 and *Parazoanthus* sp. 3. Until now, only two sponge-associated *Parazoanthus* species has been formally described from the Pacific, and none from sub-tropical or tropical waters. Five other species, *Hydrozoanthus* sp. 1, *Hydrozoanthus* sp. 2, Parazoanthidae sp. 1, Parazoanthidae sp. 2, and *Terrazoanthus* sp. 1, are also very likely to be undescribed species, but with only photographic records, or one or two specimens existing for these species, additional specimens and molecular data are needed to properly describe them.

## Conclusions

In conclusion, this study provides a starting point for zoantharian research in the Coral Triangle. We were able to discern 24 different morphological species based on specimen examination combined with photographic records. However, based on recent previous research, phylogenetic analyses of specimens would likely result in somewhat different results due to both high levels of intraspecific morphological variation in some species (Burnett et al. 1997, Reimer et al. 2004) and also morphological convergence between other phylogenetically distinct species (Sinniger and Haussermann 2009). These previous studies demonstrate how difficult it often is to properly identify zoantharian species without molecular data.

Furthermore, this study demonstrates that the central Indo-Pacific likely harbors very high levels of zoantharian diversity, as the numbers of putative species from this study (24) include a large number of undescribed species, and total numbers are as high or higher than previously reported for any other region.

Finally, it is hoped that this study can serve as a temfig for the study of other understudied coral reef benthos in the Coral Triangle. In this study, past photographic records proved to be invaluable in aiding species identification, and understanding species distributions. Thus, while museum collections should remain the key tool in taxonomic and biogeographic research (Rainbow 2009, Hoeksema et al. 2011, Rocha et al. 2014), archived in situ images can provide additional information.

## Supplementary Material

XML Treatment for Acrozoanthus australiae

XML Treatment for Zoanthus sansibaricus

XML Treatment for Zoanthus sp.

XML Treatment for Isaurus tuberculatus

XML Treatment for Neozoanthus sp.

XML Treatment for Palythoa cf. mutuki

XML Treatment for Palythoa sp.

XML Treatment for Palythoa cf. heliodiscus

XML Treatment for Palythoa aff. tuberculosa

XML Treatment for Palythoa tuberculosa

XML Treatment for Sphenopus marsupialis

XML Treatment for Sphenopus pedunculatus

XML Treatment for Hydrozoanthus gracilis

XML Treatment for Hydrozoanthus sp. 1

XML Treatment for Hydrozoanthus sp. 2

XML Treatment for Terrazoanthus sp. 1

XML Treatment for Terrazoanthus sp. 2

XML Treatment for Parazoanthidae sp. 1

XML Treatment for Parazoanthidae sp. 2

XML Treatment for Parazoanthus sp. 1

XML Treatment for Parazoanthus sp. 2

XML Treatment for Parazoanthus sp. 3

XML Treatment for Epizoanthus illoricatus

XML Treatment for Epizoanthus aff. illoricatus
